# A Polygenic Risk Score Based on a Cardioembolic Stroke Multitrait Analysis Improves a Clinical Prediction Model for This Stroke Subtype

**DOI:** 10.3389/fcvm.2022.940696

**Published:** 2022-07-08

**Authors:** Jara Cárcel-Márquez, Elena Muiño, Cristina Gallego-Fabrega, Natalia Cullell, Miquel Lledós, Laia Llucià-Carol, Tomás Sobrino, Francisco Campos, José Castillo, Marimar Freijo, Juan Francisco Arenillas, Victor Obach, José Álvarez-Sabín, Carlos A. Molina, Marc Ribó, Jordi Jiménez-Conde, Jaume Roquer, Lucia Muñoz-Narbona, Elena Lopez-Cancio, Mònica Millán, Rosa Diaz-Navarro, Cristòfol Vives-Bauza, Gemma Serrano-Heras, Tomás Segura, Laura Ibañez, Laura Heitsch, Pilar Delgado, Rajat Dhar, Jerzy Krupinski, Raquel Delgado-Mederos, Luis Prats-Sánchez, Pol Camps-Renom, Natalia Blay, Lauro Sumoy, Rafael de Cid, Joan Montaner, Carlos Cruchaga, Jin-Moo Lee, Joan Martí-Fàbregas, Israel Férnandez-Cadenas

**Affiliations:** ^1^Stroke Pharmacogenomics and Genetics Group, Institut d'Investigació Biomèdica Sant Pau (IIB SANT PAU), Barcelona, Spain; ^2^Departament de Medicina, Universitat Autònoma de Barcelona, Barcelona, Spain; ^3^Stroke Unit, Department of Neurology, Hospital de la Santa Creu i Sant Pau, Barcelona, Spain; ^4^Stroke Pharmacogenomics and Genetics Laboratory, Fundación Docència i Recerca Mútua Terrassa, Hospital Mútua Terrassa, Terrassa, Spain; ^5^Department de Genética i de Microbiologia, Universitat Autónoma de Barcelona, Barcelona, Spain; ^6^Clinical Neurosciences Research Laboratory, Hospital Clínico Universitario de Santiago de Compostela, Health Research Institute of Santiago de Compostela (IDIS), Santiago de Compostela, Spain; ^7^Department of Neurology, Biocruces-Bizkaia Health Research Institute, Bilbao, Spain; ^8^Stroke Unit, Department of Neurology, University Hospital of Valladolid, Valladolid, Spain; ^9^Department of Neurology, Hospital Clínic de Barcelona, IDIBAPS, Barcelona, Spain; ^10^Stroke Unit, Department of Neurology, Hospital Universitari Vall d'Hebron, Barcelona, Spain; ^11^Department of Neurology, IMIM-Hospital del Mar; Neurovascular Research Group, IMIM (Institut Hospital del Mar d'Investigacions Mèdiques), Universitat Autònoma de Barcelona/DCEXS-Universitat Pompeu Fabra, Barcelona, Spain; ^12^Department of Neurosciences, Hospital Germans Trias i Pujol, Universitat Autònoma de Barcelona, Barcelona, Spain; ^13^Department of Neurology, University Hospital Central de Asturias (HUCA). Oviedo, Spain; ^14^Department of Neurology, Son Espases University Hospital, Illes Balears Health Research Institute (IdISBa), Palma, Spain; ^15^Department of Neurology, University Hospital of Albacete, Albacete, Spain; ^16^Department of Psychiatry, Washington University School of Medicine, Saint Louis, MO, United States; ^17^Department of Emergency Medicine, Washington University School of Medicine, Saint Louis, MO, United States; ^18^Department of Neurology, Washington University School of Medicine, Saint Louis, MO, United States; ^19^Neurovascular Research Laboratory, Vall d'Hebron Institute of Research, Universitat Autònoma de Barcelona, Barcelona, Spain; ^20^Neurology Service, Hospital Universitari Mútua Terrassa, Terrassa, Spain; ^21^GenomesForLife-GCAT Lab, Germans Trias i Pujol Research Institute (IGTP), Badalona, Spain; ^22^High Content Genomics and Bioinformatics Unit, Germans Trias i Pujol Research Institute (IGTP), Badalona, Spain; ^23^Institute de Biomedicine of Seville, IBiS/Hospital Universitario Virgen del Rocío/CSIC/University of Seville and Department of Neurology, Hospital Universitario Virgen Macarena, Seville, Spain; ^24^Neurogenomics and Informatics Center at Washington University in St. Louis, Saint Louis, MO, United States

**Keywords:** polygenic risk score, GWAS, multi-trait analysis, stroke, ESUs

## Abstract

**Background:**

Occult atrial fibrillation (AF) is one of the major causes of embolic stroke of undetermined source (ESUS). Knowing the underlying etiology of an ESUS will reduce stroke recurrence and/or unnecessary use of anticoagulants. Understanding cardioembolic strokes (CES), whose main cause is AF, will provide tools to select patients who would benefit from anticoagulants among those with ESUS or AF. We aimed to discover novel loci associated with CES and create a polygenetic risk score (PRS) for a more efficient CES risk stratification.

**Methods:**

Multitrait analysis of GWAS (MTAG) was performed with MEGASTROKE-CES cohort (*n* = 362,661) and AF cohort (*n* = 1,030,836). We considered significant variants and replicated those variants with MTAG *p*-value < 5 × 10^−8^ influencing both traits (GWAS-pairwise) with a *p*-value < 0.05 in the original GWAS and in an independent cohort (*n* = 9,105). The PRS was created with PRSice-2 and evaluated in the independent cohort.

**Results:**

We found and replicated eleven loci associated with CES. Eight were novel loci. Seven of them had been previously associated with AF, namely, *CAV1, ESR2, GORAB, IGF1R, NEURL1, WIPF1*, and *ZEB2*. *KIAA1755* locus had never been associated with CES/AF, leading its index variant to a missense change (R1045W). The PRS generated has been significantly associated with CES improving discrimination and patient reclassification of a model with age, sex, and hypertension.

**Conclusion:**

The loci found significantly associated with CES in the MTAG, together with the creation of a PRS that improves the predictive clinical models of CES, might help guide future clinical trials of anticoagulant therapy in patients with ESUS or AF.

## Introduction

About 25% of ischemic strokes are of undetermined etiology (1): patients with multiple stroke etiologies, incomplete diagnostic work-up, or embolic stroke of undetermined source (ESUS). Up to 17% of all ischemic strokes are ESUS, with a stroke recurrence rate of 4–5% despite antiplatelet therapy (2).

The ESUS encompasses different entities. Atrial cardiopathy, occult atrial fibrillation (AF), and left ventricular disease might benefit from anticoagulation, but atherosclerotic plaques might benefit from low-dose anticoagulation with antiplatelets in ESUS patients (2). The subgroup of patients >75 years in RE-SPECT ESUS (Dabigatran Etexilate for Secondary Stroke Prevention in Patients With Embolic Stroke of Undetermined Source) had a significant benefit of lower-dose dabigatran over aspirin, suggesting occult AF as a triggering cause (3). Different studies indicate that the prevalence of occult AF among ESUS patients is 11–30% (2, 4).

A tool capable of better stratifying patients is needed to offer them appropriate treatment regarding its potential stroke cause to decrease its recurrence.

On the contrary, not all patients with AF will develop a stroke, and the decision of anticoagulation for stroke prevention in AF patients is carried out based on a clinical scale: CHA_2_DS_2_-VASc. The rates of stroke vary considerably in patients with CHA_2_DS_2_-VASc 1–2 (5) and hence the need for a more accurate scale in these cases.

Cardioembolic strokes (CES) are mostly caused by an onset/already known AF. Understanding CES genetic architecture will provide tools to select ESUS or AF patients who would benefit from anticoagulants and develop specific and more effective therapies with fewer side effects.

Therefore, we aimed to discover novel loci associated with CES by performing a Multitrait Analysis of Genome Wide Association Study (MTAG) of CES-AF and create a polygenetic risk score (PRS) that allowed a more efficient stratification of stroke patient risk of having a CES.

## Methods

The data that support the findings of this study are available from the corresponding author upon reasonable request.

### Cohorts' Description

The summary statistics for CES were obtained from the MEGASTROKE analysis (MEGASTROKE-CES) through the Cerebrovascular Disease Knowledge Portal (http://cerebrovascularportal.org). This cohort was composed of 7,193 CES patients and 355,468 controls of European ancestry. The summary statistics for AF were obtained from the Atrial Fibrillation 2018 (AF-2018) analysis through the GWAS catalog portal (https://www.ebi.ac.uk/gwas/). The AF-2018 cohort was composed of 60,620 AF cases and 970,216 controls. The characteristics of the individuals in both studies are listed in the Supplementary Material and their respective publications (6, 7).

### Single-Nucleotide Variation Quality Controls

A series of standard quality controls (QC) was applied to select the single-nucleotide variants (SNVs) for the analysis. Variant exclusion criteria include the following (1): Not common to the summary statistics of the traits (2), Minor allele frequency lower or equal to 0.01 (3), Missing values (4), Negative standard error or not a number value (5), *p*-value of 0, 6 Not SNVs (7), Duplicated SNVs (8), Strand ambiguity, and (8) Inconsistent allele pairs. Locus 15q21.3, which prioritized genes *GCOM1* and *MYZAP* from AF-2018, was not evaluated due to the absence of the significant SNVs of AF-2018 in the MEGASTROKE-CES analysis.

### Multitrait Analysis of GWAS

We applied MTAG (8) of MEGASTROKE-CES and AF-2018 summary statistics. We considered loci to be significantly associated with the trait of interest when the *p*-value was < 5 × 10^−8^ in the MTAG result and the *p*-value was < 0.05 in the original GWAS. We considered replicating the SNVs with a *p*-value < 0.05 in the GWAS of our independent cohort.

To avoid an increase in the type I error rate due to the presence of SNVs that are not associated with CES but with AF or vice versa, we used GWAS-pairwise (9). This is a Bayesian pleiotropy association test to identify genetic variants that influence pairs of traits (9). We used it to ensure that the leading SNV of a significant locus belongs to a genomic region influenced by both traits evaluated (9), since SNPs that are not really associated with one trait, but are associated with the other one, could bias effect-size estimates for the first trait and increase false-positive rate (8). The posterior probability for model-3 (PPA-3) >0.6 suggests that a specific genomic region is associated with both traits. A PPA-1 >0.6 will suggest that the genomic region is associated only with CES, and a PPA-2 >0.6 is associated only with AF. Genomic inflation was estimated as lambda.

### Identification of Independent and Novel Loci Associated With CES

Independent loci were defined as those >1 megabase (Mb) apart in the physical distance among SNVs with a genome-wide significance threshold of *p*-value < 5 × 10^−86^. Loci were defined as novel when SNVs had an *r*^2^ < 0.1 compared with the index SNVs of the loci, namely, *PITX2* ^7^, *ZFHX3* ^7^, *NKX2-5* ^7^, *RGS7* ^7^, *ABO* ^7^ ^10^, *PHF20* ^11^, *GNAO1* ^11^, and 5q22.3 ^11^, that were GWAS significant in previous studies.

### Replication Stage in an Independent European Cohort

We performed GWAS in an independent cohort of 9,105 individuals [GENERACION cohort: 3,479 ischemic stroke (IS) patients and 5,625 controls]. IS patients over 18 years were recruited *via* hospital-based studies, between 2003 and 2020 in Spain, if they had a measurable neurologic deficit on the NIHSS within 6 h of the last known asymptomatic status and had been diagnosed with stroke by an experienced neurologist and confirmed by neuroimaging (10, 11). Controls were subjects over 18 years recruited in Spain, without a history of IS, who declared they were free of neurovascular diseases before enrollment. An Institutional Review Board or Ethics Committee approved the study at each participating site. All patients or their relatives provided written informed consent. Further description of the cohorts is present in Supplementary Material, as well as the array information, the contribution of hospitals, and the clinical description (Supplementary Tables 1–3).

#### Quality Control and Imputation

The DNA samples were genotyped on commercial arrays from Illumina® (San Diego, CA) and Axiom^TM^ Spain Biobank array (Supplementary Table 2). Standard QCs were performed using the PLINK v1.9 and KING v2.1.3 software. Imputation was performed in the Michigan Imputation Server Pipeline (12) using Minimac4 and HRC r1.1 2016 panel. Further descriptions of QCs and imputation are present in the Supplementary Material.

#### GWAS Analysis

We performed two different GWAS in the same cohort (for the two different traits here studied), with an additive genetic model using fastGWA from GCTA (13). We studied the association with CES (CES = 1,515; controls = 5,626) and AF (AF patients = 1,110; controls = 7,791). Age, sex, and the first 10 principal components were used as covariates.

The results of these two GWAS were used to evaluate replicability. We studied those index variants from significant loci with a *p*-value < 5 × 10^−8^ in the MTAG, a *p*-value < 0.05 in the original GWAS used for performing the MTAG, and PPA-3 > 0.6 that suggests that the genomic region is associated with CES and AF. We considered the replicated SNVs with a *p*-value < 0.05 and a consistent direction of the effect on this analysis.

### Functional Annotation and Gene Prioritization

Gene prioritization was performed for the novel loci using Variant-to-Gene tool from Open targets Genetics Version 7 (14). This tool integrates biological evidence of four main data types, namely, (1) molecular phenotype quantitative trait loci experiments (QTLs), (2) chromatin interaction experiments, e.g., Promoter Capture Hi-C (PCHi-C), 3) *in silico* functional predictions, e.g., Variant Effect Predictor (VEP) from Ensembl and (4) distance between the variant and each gene's canonical transcription start site (TSS). Additionally, we used the HaploReg database to determine the functional annotation of the most strongly associated SNVs per locus. For the missense SNVs, we determined the likelihood that amino acid substitution has a deleterious effect on protein function using SIFT.

### Gene Set Analysis

We conducted a WebGestalt Overrepresentation Analysis of the selected prioritized genes associated with MTAG-CES. Gene Ontology (GO) of biological processes was performed, as well as a Benjamini Hochberg correction of the association *p*-value. We defined a biological process with a *p*-value < 0.05 as statistically significant.

### Polygenic Risk Score Development

A PRS was conducted through the PRSice-2 software version 2.3.3 (15), where the estimation is based on the risk alleles of having a CES and their effect size extracted from the regions with PPA-3 > 0.6 of the MTAG summary statistics created in this study.

GENERACION cohort was randomly split into training and test sets in 80:20 proportion. Best score threshold selection was performed based on the major variance explained by the score (PRS r^2^) in the training set. The evaluation of this score was performed in the independent test set.

We used R version 4.1.3 and Bioconductor packages to evaluate the clinical relevance of this PRS. We calculated three models, namely, model-1 including only the PRS; model-2 including statistically significant clinical variables with <10% missing values, since a high rate of missing values might bias the results of subsequent statistical analyses (16); and model-3 adding the PRS to the model-2. Model discrimination was assessed with the area under the ROC curve (AUC) and the area under the precision recall curve (AUPRC). We used DeLong's test for two correlated ROC curves to find out whether there are significant differences between the discrimination of the models. The net reclassification index (NRI) and integrated discrimination index (IDI) were performed to evaluate model-2 and model-3. Additionally, we estimated the AUC and AUPRC for each individual predictor.

## Results

### MTAG Analysis of CES

**Figure 1:**
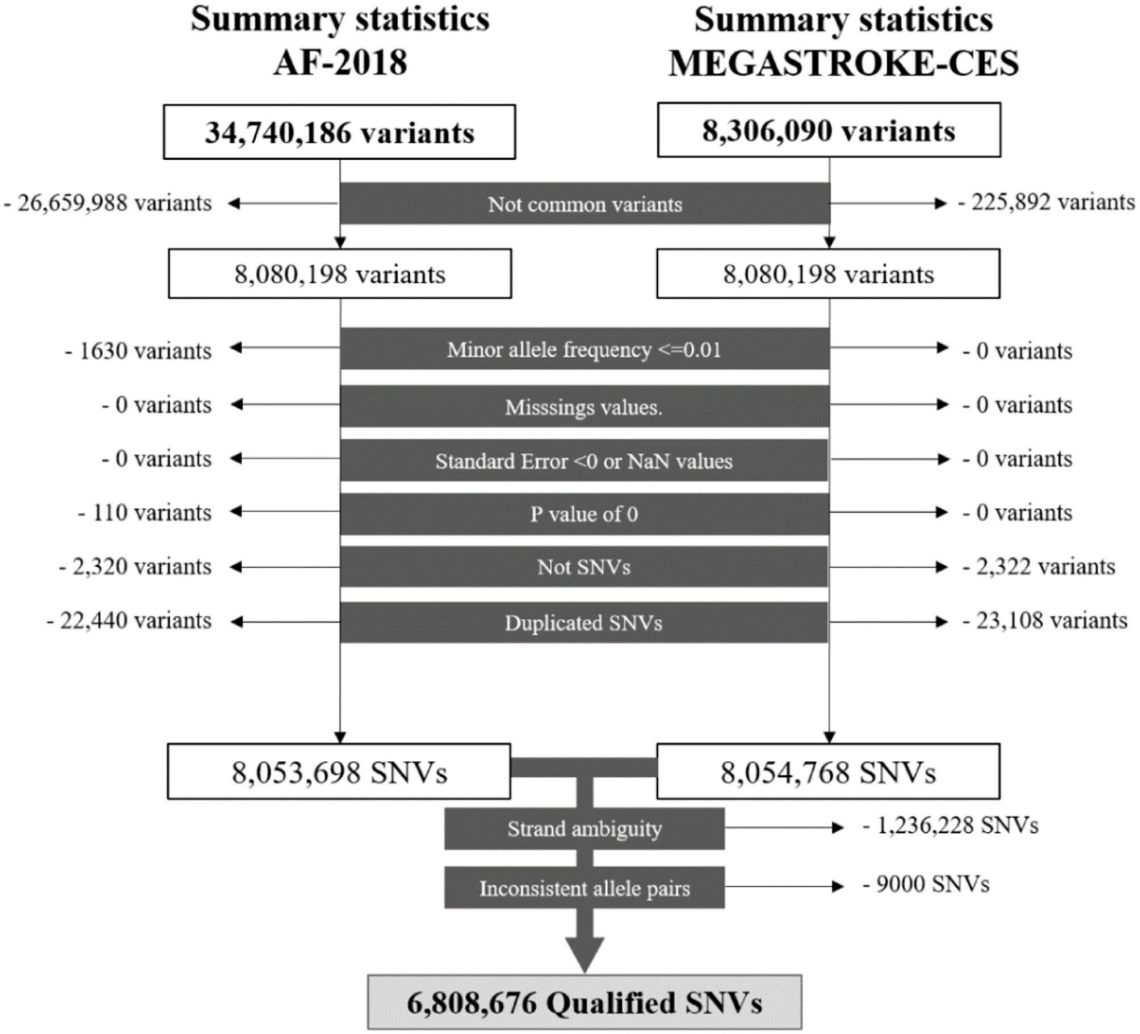
Workflow of the SNVs for the AF-2018 and MEGASTROKE-CES datasets. NaN, Not a number; SNV, Single-nucleotide variant.

**Figure 2:**
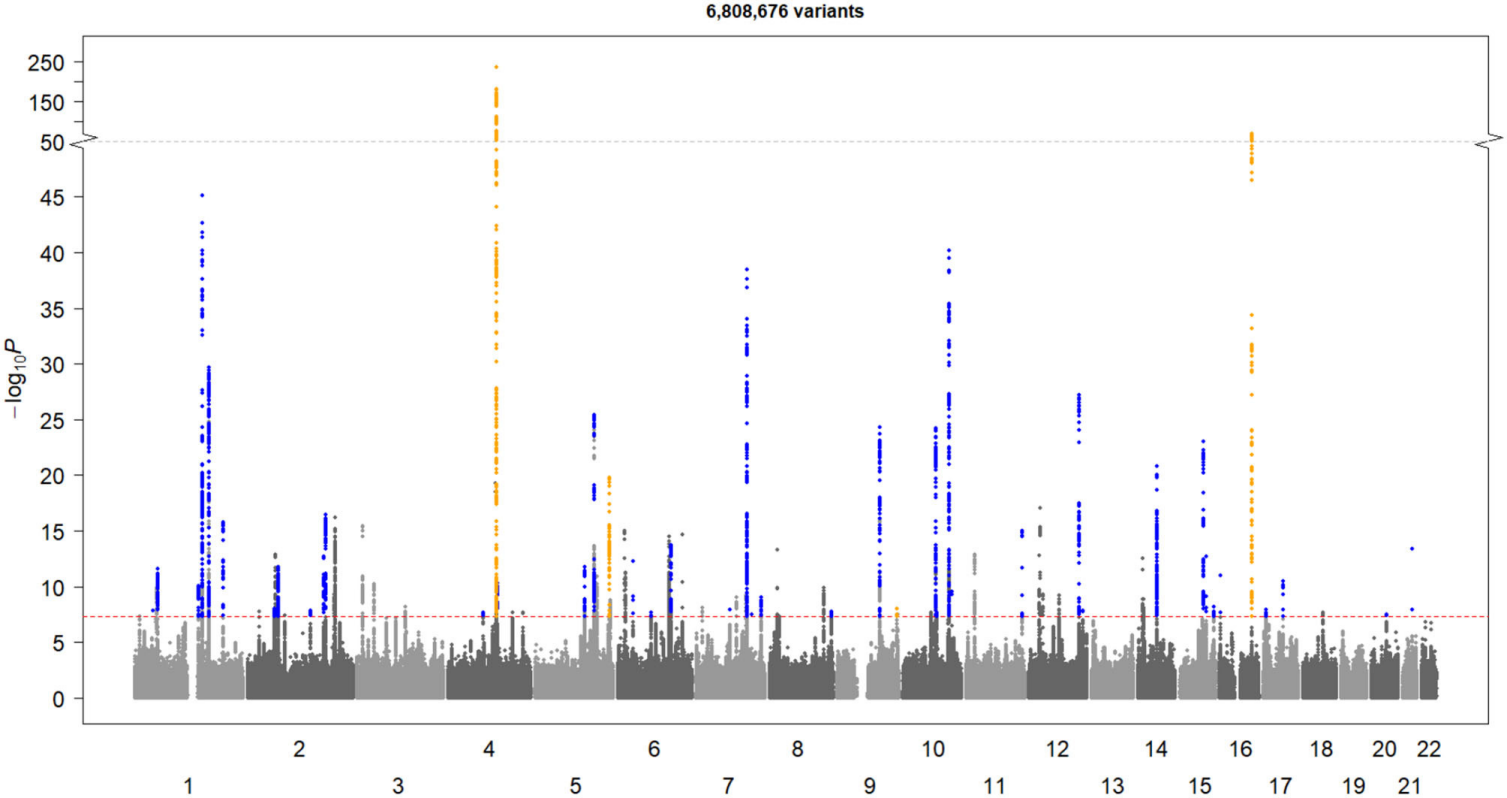
Manhattan plot of MTAG-CES. The X-axis represents chromosome location, and the Y-axis represents the minus logarithm on base 10 of *p*-value. The red line represents the GWAS-significance threshold. The novel loci are shown in blue and the established loci are shown in yellow.

After QCs (Figure 1), there were 6,808,676 common qualified SNVs from the AF-2018 and MEGASTROKE-CES cohorts. The MTAG software revealed mean χ^2^ for AF-2018 and MEGASTROKE-CES in 1.39 and 1.12, respectively. The estimated equivalent GWAS sample size of the MTAG analysis for CES was 861,823 individuals. A Manhattan plot of the MTAG-CES analysis is shown in Figure 2; less evidence of genomic inflation was observed with a lambda of 1.02.

The MTAG-CES results revealed a total of 44 associated loci (*p*-value < 5 × 10^−8^); 40 significant loci associated with CES were novel, and four were previously found known associations (Table 1). All loci significantly associated with MEGASTROKE-CES (*ABO, NKX2-5, PITX2*, and *ZFHX3*) were genome-wide associated in this MTAG-CES, except for the locus belonging to *RGS7* gene (top SNV rs146390073 MTAG *p*-value = 0.001, AF-2018 *p*-value = 0.98).

**Table 1:** MTAG-CES results of the independent and significant loci.

				**MEGASTROKE CES**		**AF-2018**		**MTAG-CES**		**MTAG-AF**		
**SNV**	**Locus**	**Gene**	**Novelty**	**Z**	**P**	**Z**	**P**	**Z**	**P**	**Z**	**P**	**PPA**
rs17042098-A	4q25	*PITX2*	(Malik et al.) [7]	12.72	3.72 x 10^−37^	38.01	8.97 x 10^−318^	32.86	7.73 x 10^−237^	37.93	0	1.00
rs2106261-T	16q22.3	*ZFHX3*	(Malik et al.) [7]	6.75	1.63 x 10^−11^	20.23	4.97 x 10^−91^	17.48	2.02 x 10^−68^	20.19	1.20 x 10^−90^	1.00
rs11264280-T	1q21.3	*ADAM15*	Novel	2.49	1.28 x 10^−02^	18.97	3.07 x 10^−79^	14.22	7.17 x 10^−46^	18.44	5.88 x 10^−76^	0.65
rs11598047-A	10q24.33	*NEURL1*	Novel	−3.27	1.06 x 10^−03^	−17.08	8.95 x 10^−66^	−13.38	7.50 x 10^−41^	−16.73	7.42 x 10^−63^	0.93
rs3807989-A	7q31.2	*CAV1*	Novel	−4.50	6.75 x 10^−06^	−15.63	1.24 x 10^−54^	−13.10	3.43 x 10^−39^	−15.50	3.30 x 10^−54^	1.00
rs680084-A	1q24.2	*GORAB*	Novel	−4.10	4.02 x 10^−05^	−13.54	3.31 x 10^−42^	−11.46	2.10 x 10^−30^	−13.46	2.84 x 10^−41^	0.99
rs883079-T	12q24.21	*TBX5*	Novel	3.55	3.95 x 10^−04^	13.26	2.84 x 10^−40^	10.96	5.97 x 10^−28^	13.12	2.60 x 10^−39^	0.97
rs17171711-T	5q31.2	*FAM13B*	Novel	3.79	1.56 x 10^−04^	12.48	1.95 x 10^−35^	10.57	4.04 x 10^−26^	12.41	2.35 x 10^−35^	0.99
rs4385527-A	9q22.32	*AOPEP*	Novel	3.92	8.56 x 10^−05^	12.03	6.16 x 10^−33^	10.34	4.69 x 10^−25^	11.99	3.89 x 10^−33^	0.95
rs78249997-T	10q22.2	*MYOZ1*	Novel	−3.82	1.37 x 10^−04^	−12.08	8.75 x 10^−34^	−10.32	5.80 x 10^−25^	−12.03	2.45 x 10^−33^	0.98
rs7172038-T	15q24.1	*NEO1*	Novel	−2.73	6.43 x 10^−03^	−12.58	4.78 x 10^−36^	−10.04	1.02 x 10^−23^	−12.37	3.76 x 10^−35^	0.74
rs2738413-A	14q23.2	*ESR2*	Novel	2.97	3.03x10^−03^	11.61	2.55 x 10^−31^	9.52	1.67 x 10^−21^	11.47	1.80 x 10^−30^	0.85
rs6891790-T	5q35.1	*NKX2-5*	(Malik et al.) [7]	−4.97	6.67x10^−07^	−9.59	4.53 x 10^−22^	−9.29	1.57 x 10^−20^	−9.80	1.16 x 10^−22^	1.00
rs2857265-A	2q31.2	*FKBP7*	Novel	2.76	5.85 x 10^−03^	10.16	4.58 x 10^−24^	8.42	3.65 x 10^−17^	10.06	8.29 x 10^−24^	0.70
rs10753933-T	1q32.1	*PPFIA4*	Novel	3.72	2.09 x 10^−04^	9.09	9.84 x 10^−20^	8.24	1.70 x 10^−16^	9.16	5.30 x 10^−20^	0.99
rs74399915-T	11q24.3	*C11orf45*	Novel	3.38	7.20 x 10^−04^	9.05	1.23 x 10^−19^	8.03	1.01 x 10^−15^	9.08	1.11 x 10^−19^	0.92
rs13191450-A	6q22.31	*HSF2*	Novel	2.93	3.51 x 10^−03^	8.88	9.97 x 10^−19^	7.65	1.95 x 10^−14^	8.86	7.96 x 10^−19^	0.81
rs2834618-T	21q22.12	*RUNX1*	Novel	3.31	9.48 x 10^−04^	8.43	3.41x 10^−17^	7.56	3.96 x 10^−14^	8.47	2.37 x 10^−17^	0.96
rs56181519-T	2q31.1	*WIPF1*	Novel	−2.73	6.31 x 10^−03^	−8.60	6.46 x 10^−18^	−7.35	1.98 x 10^−13^	−8.56	1.11 x 10^−17^	0.82
rs12908004-A	15q25.1	*ARNT2*	Novel	−3.28	1.03 x 10^−03^	−8.13	4.12 x 10^−16^	−7.35	2.03 x 10^−13^	−8.19	2.65 x 10^−16^	0.96
rs3176326-A	6p21.2	*CDKN1A*	Novel	−3.98	6.66 x 10^−05^	−7.36	1.42 x 10^−13^	−7.23	5.01 x 10^−13^	−7.54	4.59 x 10^−14^	1.00
rs6747542-T	2p13.3	*GMCL1*	Novel	2.61	8.86 x 10^−03^	8.27	1.10 x 10^−16^	7.06	1.63 x 10^−12^	8.23	1.82 x 10^−16^	0.75
rs337705-T	5q22.3	*KCNN2*	Novel	−2.56	1.04 x 10^−02^	−8.29	1.63 x 10^−16^	−7.05	1.77 x 10^−12^	−8.25	1.57 x 10^−16^	0.72
rs41292535-A	1p32.2	*EPS15*	Novel	3.81	1.39 x 10^−04^	7.16	7.42 x 10^−13^	6.99	2.73 x 10^−12^	7.33	2.34 x 10^−13^	0.96
rs140185678-A	16p13.3	*RPL3L*	Novel	2.92	3.54 x 10^−03^	7.61	2.43 x 10^−14^	6.79	1.13 x 10^−11^	7.64	2.13 x 10^−14^	0.89
rs76774446-A	17q21.32	*GOSR2*	Novel	4.38	1.20 x 10^−05^	6.15	8.72 x10^−10^	6.63	3.32 x 10^−11^	6.43	1.25 x 10^−10^	0.99
rs55754224-T	4q26	*CAMK2D*	Novel	2.80	5.05 x 10^−03^	7.39	2.15 x 10^−13^	6.57	4.92 x 10^−11^	7.41	1.22 x 10^−13^	0.80
rs79187193-A	1q21.2	*GJA5*	Novel	−2.37	1.78 x 10^−02^	−7.59	3.15 x 10^−14^	−6.47	9.79 x 10^−11^	−7.56	4.08 x 10^−14^	0.71
rs12260801-T	10q25.2	*PDCD4*	Novel	4.56	5.22 x 10^−06^	5.53	2.94 x 10^−08^	6.31	2.79 x 10^−10^	5.86	4.62 x 10^−09^	1.00
rs2269001-A	7q36.1	*KCNH2*	Novel	−2.89	3.94 x 10^−03^	−6.63	4.01 x 10^−11^	−6.11	1.00 x 10^−09^	−6.70	2.06 x 10^−11^	0.73
rs6598541-A	15q26.3	*IGF1R*	Novel	2.69	7.17 x 10^−03^	6.35	2.22 x 10^−10^	5.81	6.21 x 10^−09^	6.41	1.48 x 10^−10^	0.72
rs11125871-T	2p15	*C2orf74*	Novel	−3.24	1.17 x 10^−03^	−5.79	6.42 x 10^−09^	−5.75	9.19 x 10^−09^	−5.95	2.71 x 10^−09^	0.87
rs635634-T	9q34.2	*ABO*	(Williams et al.) [10]; Malik et al. [7]	5.10	3.31 x 10^−07^	4.20	2.74 x 10^−05^	5.72	1.04 x 10^−08^	4.66	3.13 x 10^−06^	1.00
rs10272350-A	7q11.23	*TMEM60*	Novel	4.53	6.13 x 10^−06^	4.62	3.41 x 10^−06^	5.68	1.32 x 10^−08^	4.99	5.93 x 10^−07^	0.99
rs116600817-A	17p13.1	*TNFSF12*	Novel	2.88	3.92 x 10^−03^	6.00	2.45 x 10^−09^	5.68	1.33 x 10^−08^	6.10	1.07 x 10^−09^	0.85
rs13010313-T	2q22.3	*ZEB2*	Novel	3.52	4.21 x 10^−04^	5.45	5.72 x 10^−08^	5.67	1.41 x 10^−08^	5.65	1.57 x 10^−08^	0.90
rs2885697-T	1p34.2	*SCMH1*	Novel	−2.54	1.09 x 10^−02^	−6.27	2.88 x 10^−10^	−5.67	1.41 x 10^−08^	−6.32	2.70 x 10^−10^	0.72
rs11057583-A	12q24.31	*NCOR2*	Novel	3.82	1.35 x 10^−04^	5.19	2.10 x 10^−07^	5.67	1.46 x 10^−08^	5.45	5.13 x 10^−08^	0.74
rs11782313-T	8q24.3	*PTK2*	Novel	−3.05	2.34 x 10^−03^	−5.81	5.71 x 10^−09^	−5.64	1.65 x 10^−08^	−5.94	2.94 x 10^−09^	0.78
rs12211255-A	6q14.1	*FILIP1*	Novel	3.42	6.24 x 10^−04^	5.44	5.06 x 10^−08^	5.61	2.06 x 10^−08^	5.63	1.78 x 10^−08^	0.96
rs1898096-A	10q22.3	*LRMDA*	Novel	−2.64	8.30 x 10^−03^	−6.08	1.39 x 10^−09^	−5.60	2.13 x 10^−08^	−6.15	7.96 x 10^−10^	1.00
rs11099098-T	4q21.21	*FGF5*	Novel	2.77	5.67 x1 0^−03^	5.94	2.96 x 10^−09^	5.58	2.38 x 10^−08^	6.03	1.62 x 10^−09^	0.78
rs55985730-T	7q32.1	*CALU*	Novel	−2.84	4.47 x 10^−03^	−5.82	5.24 x 10^−09^	−5.54	3.06 x 10^−08^	−5.92	3.19 x 10^−09^	0.87
rs3746471-A	20q11.23	*KIAA1755*	Novel	3.56	3.58 x 10^−04^	5.18	2.30 x 10^−07^	5.51	3.50 x 10^−08^	5.40	6.59 x 10^−08^	0.96

*SNV, Index Single Nucleotide Variant and assessed allele; Z, Z-score; P, P-value. Loci highlighted in bold are the ones not previously associated with AF. PPA, posterior probability of model 3 of GWAS-PW*.

Other loci found significant in previous GWAS of CES different from MEGASTROKE were: *PHF20, GNAO1*, and 5q22.3 region. For two of them, the association was more significant in our analysis. For the 5q22.3 region, top SNV rs2169955 MTAG-CES *p*-value = 4.76 × 10^−7^ vs. MEGASTROKE-CES *p*-value = 6.13 × 10^−3^, and for mapped gene *PHF20*, top SNV rs11697087 MTAG-CES *p*-value = 6.62 × 10^−5^ vs. MEGASTROKE-CES *p*-value = 6.05 × 10^−4^. *GNAO1* was not evaluated in our study due to the absence of the index SNV in AF-2018.

### New Candidate Loci Associated With CES

After gene prioritization, 44 genes were selected from the 44 loci (Supplementary Table 4). Novel loci showed a high degree of functionality of the SNVs as missense variants, eQTL, pQTLs, and HiC physical interaction (Supplementary Table 4).

Replication analysis was performed in a new cohort of IS patients and controls (GENERACION cohort, *n* = 9,105). Evaluation of the index SNVs and SNVs in high LD belonging to genome-wide significant loci from the MTAG-CES revealed that 11 loci were replicated, as SNVs had a *p*-value < 0.05 in MEGASTROKE-CES, a PPA-3 > 0.6, and a *p*-value < 0.05 in the replication cohort (GENERACION). Among these 11 different loci, *PITX2, ZFHX3*, and *NKX2-5* were already known. Eight loci were novel associations whose prioritized genes were *CAV1, IGF1R, KIAA1755, NEURL1, GORAB, ESR2, ZEB2*, and *WIPF1* (Supplementary Table 5).

Interestingly, we found loci not previously described in AF or CES with four prioritized genes, namely, *TMEM60, KIAA1755, NCOR2*, and *FILIP1*. Functional annotation of the index SNVs revealed rs3746471 as a missense variant of the *KIAA1755* gene coding for R1045W, and it was predicted to be deleterious with a SIFT score of 0.007.

### New Candidate Locus Associated With AF

*FILIP1* locus reached genome-wide significance in the MTAG-AF, a *p*-value < 0.05 in AF-2018 and a PPA-3 > 0.6. The FILIP1 index variant was additionally evaluated in the GWAS of AF in the independent cohort (GENERACION), revealing a suggestive *p*-value with a consistent direction of the effect of this novel association with AF rs12211255-A, beta(se) = 0.013(0.007), *p*-value = 0.09.

The study of the 111 AF-2018 significant loci (Supplementary Table 6) using GWAS-pairwise strategy suggested 51 loci that have an exclusive association with AF risk and a lack of association with CES.

### Biological Processes of Loci Associated With CES and AF and Biological Processes of Loci Associated Exclusively With AF

The GO of biological processes from the Genome-Wide loci of the MTAG-CES analysis revealed 98 enriched gene sets (Supplementary Figure 1, Supplementary Table 7); the top biological processes were cardiac conduction, cardiac muscle cell contraction, and cardiac muscle contraction.

A biological process analysis of the genes associated exclusively with AF (Supplementary Figure 2, Supplementary Table 8) revealed 41 biological processes exclusive to AF risk and mainly associated with cardiac development processes (Supplementary Figure 1, Supplementary Table 9).

### Polygenic Risk Score

The training set was composed of 1,212 CES patients and 4,501 controls and the test set of 303 CES patients and 1,125 controls from GENERACION. No significant differences in clinical variables were found between the training and the test sets (Supplementary Table 10).

**Figure 3:**
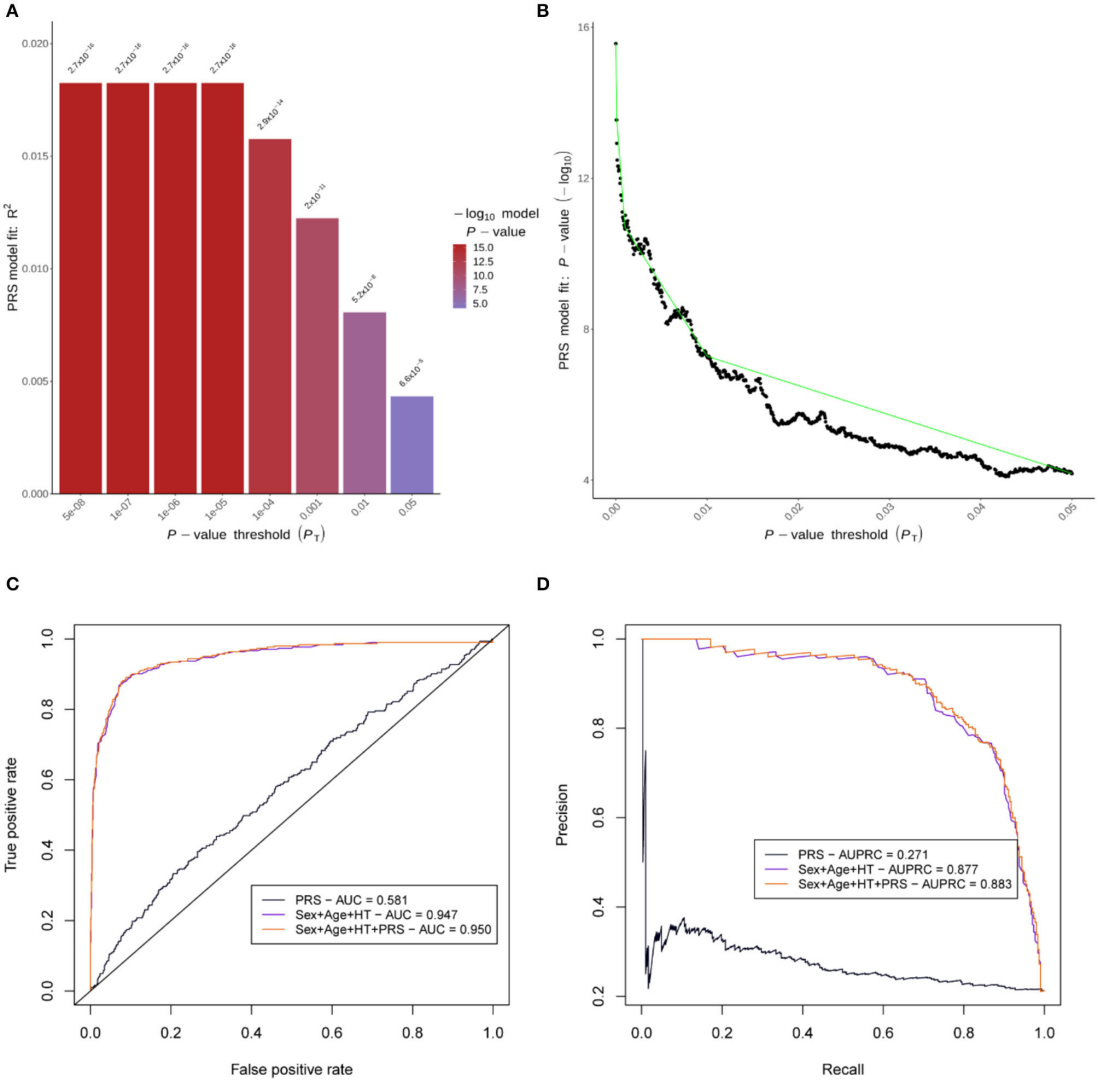
Polygenic risk score (PRS) performance. **(A)** is a bar plot of the *r*^2^ for the PRS models of eight different thresholds in the training set. **(B)** represents the *p*-value variation along the full range of thresholds evaluated in the training set. **(C)** shows ROC curves, and **(D)** shows precision-recall curves for the PRS performance in the independent test set. AUC, area under the ROC curve; AUPRC, area under the precision-recall curve; HT, hypertension.

For model-1, the PRS with the highest *r*^2^ in the training set (*r*^2^ = 0.018) was obtained with an SNV *p*-value threshold of 5 × 10^−8^, comprising a total of 93 SNVs (Figure 3). Age, sex, and hypertension were the only variables for which information was available for >90% of the patients, and therefore, the only ones considered in the multivariable model as mentioned in the Methods” section. The three variables were significantly associated and therefore included in model-2. For model-3, we added the PRS to model-2, and all remaining variables were significant (Supplementary Table 11), including the PRS with a *Z*-value of 4.33 and a *p*-value of 1.28 × 10^−5^.

The AUC in the test set for the different models was 0.581 in model-1, 0.947 in model-2, and 0.950 in model-3. AUPRC was 0.271 in model-1, 0.877 in model-2, and 0.883 in model-3 (Figure 3). Comparing AUC, there was significantly better discrimination in model-3 than model-2 (*Z*-score = −2.50, *p*-value = 0.01). AUC and AUPRC for each individual predictor can be found in Supplementary Figure 3.

Additionally, the NRI categorical and quantitative and IDI showed a significant reclassification when quartiles of score risk were analyzed (Table 2).

**Table 2:** Reclassification table comparing CES models with and without PRS addition.

	**Risk Category Age+Sex+HT+PRS model**				
**Risk Category Age+Sex+HT model**	**No**.	**No**.	**No**.	**No**.	**% Reclassified**
*Non cases*					
Q1	4,062	23	0	0	1
Q2	25	174	16	0	19
Q3	0	23	120	4	18
Q4	0	0	3	50	0
*Cases*					
Q1	149	7	0	0	4
Q2	7	124	10	0	12
Q3	0	12	171	34	21
Q4	0	0	18	677	3
NRI (Categorical) [95% CI]: 0.0134 [−0.0024–0.0291]; *p*-value: 0.09688		
NRI (Continuos) [95% CI]: 0.1416 [0.0782–0.2049]; *p*-value: 1e-5		
IDI [95% CI]: 0.0029 [9e-04−0.0049]; *p*-value: 0.00431			

*Q, quartile; NRI, Net Reclassification Index; IDI, integrated discrimination improvement*.

## Discussion

Using an MTAG with the two biggest cohorts of CES (7) and AF (6) to date, we found 44 genome-wide significant loci associated with CES. The prioritized genes of this loci were involved in biological processes such as cardiac conduction and contraction. Nevertheless, the 51 loci associated exclusively with AF (not associated with CES as shown in the GWAS-pairwise) were mainly associated with cardiac development processes. This highlights the possible role in the risk of stroke due to AF of genes related to cardiac conduction and contraction instead of the cardiac development process and thereby would help to develop more specific prevention drugs.

Eleven loci significantly associated with CES were replicated in the independent cohort. Their prioritized genes are listed as follows: *PITX2, ZFHX3, NKX2-5, CAV1, IGF1R, KIAA1755, NEURL1, GORAB, ESR2, ZEB2*, and *WIPF1*. Of the genes associated with these loci, *PITX2, ZFHX3*, and *NKX2-5* were already known to be associated with CES and AF. Eight were new CES associations; seven of them were previously associated with AF, namely, *CAV1, ESR2, GORAB, IGF1R, NEURL1, WIPF1*, and *ZEB2*; and KIAA1755 was a completely new association with CES, not being previously associated with AF.

One could think that by increasing the statistical power to find CES-associated SNVs through enrichment of AF patients, part of the associations is due to actually being associated only with AF. For this reason, we ensure that SNVs belonged to genomic regions associated with AF and CES through GWAS-pairwise (PPA-3 > 0.6). Therefore, these 11 SNVs could be markers of stroke risk among patients with ESUS or among AF patients, as they are SNVs located in genomic regions that are not exclusively associated with either CES or FA, but with both.

Of the new loci associations with CES, we could highlight some genes. *CAV1* encodes caveolin-1, the principal structural component of caveolae organelles in smooth muscle cells and endothelial cells (17). Caveolin-1 confers an anti-AF effect by mediating atrial structural remodeling through its antifibrotic action (18). Also, it plays a key role in how gas6 exerts its prothrombotic role in the vasculature (19). Genetic disruption of caveolin-1 in mice induces a severe biventricular hypertrophy with systolic and diastolic heart failure (20). That supports the relevance that caveolin-1 might have in other causes of CES as symptomatic congestive heart failure with reduced ejection fraction (21), or its importance in ESUS as a marker of an occult FA or left ventricular disfunction, which could benefit from anticoagulant treatment.

*ESR2* encodes for the estrogen receptor beta, one of the receptors that mediates the biological effects of estrogens, which increase the levels of procoagulant factors VII, IX, X, XII, and XIII and reduce the concentrations of the anticoagulant factors protein S and antithrombin (22). Therefore, it might be a stroke risk marker.

*IGF1R* encodes the insulin-like growth factor (IGF) 1 receptor, that is, the main receptor mediating IGF signaling in the heart (23). Inhibition of the IGF receptor decreases the proliferation of cardiomyocytes in murine embryonic stem cells (23). *ZEB2* encodes the zinc finger E-box-binding homeobox 2 protein that regulates cardiac fibroblast activation. An aberrant activation could lead to structural changes prone to develop AF.

*KIAA1755* has not previously been found associated with AF. The index variant of this locus, rs3746471-A, encodes for R1045W amino acid change, predicted to be deleterious according to SIFT. rs3746471-A has been previously described as associated with heart rate (24–26) and PR interval (26) and is remarkably suggestively associated with stroke infarct volume (*p*-value = 6.80 × 10^−7^) (27, 28). *KIAA1755* is predicted to encode an uncharacterized protein and is only characterized at the transcriptional level. The transcript is highly expressed in the brain and nerves and is also expressed in the heart.

We also found a novel locus suggestive to be associated with AF: 6q14.1, being *FILIP1* the prioritized gene linked with the leading SNV of the locus. This gene encodes a filamin A binding protein and has been identified as a regulator of myogenesis differentiation in human cells and in an *in vivo* mouse model (29). In the replication stage, this SNV was found suggestive (*p*-value = 0.09), highly probable due to the small sample size in comparison with MTAG analysis.

The PRS generated with the SNVs from MTAG-CES was associated with CES independently of age, sex, and hypertension, being simpler than other PRS that needs a major number of SNVs for association (30). We found that the addition of our PRS to a model with age, sex, and hypertension significantly improves the discriminatory power to detect CES.

Interestingly, the quantitative NRI was estimated in 14.16%, which is the proportion of cases correctly assigned to a higher probability of CES, among controls correctly assigned to a lower probability by an updated model adding our PRS compared with the initial model without it.

As limitations, the difference in the sample size between the two original studies could lead to significant results for SNVs that are truly null for one trait but not for another, biasing effect-size estimates for the first trait and increasing the false discovery rate (and inflated type I error rate) (8). Nevertheless, MTAG estimation of χ^2^ revealed a scenario expected to be strong against false positives, as tested in the original publication (8), and less evidence on genomic inflation was observed. Besides, we used GWAS-pairwise to ensure that the novel loci were not associated with only one of the traits, but with both at the same time, having a PPA-3 > 0.6. But even more important, as usually in this kind of studies, we validated the significant loci found in this MTAG-CES and MTAG-AF in a GWAS of an independent European cohort. The small size of this last cohort underpowers the ability to find significant results. However, we were able to replicate 11 leading SNVs from the total number of significant loci in the MTAG-CES and suggest one new potential locus in the MTAG-AF.

Another limitation is that we have only found loci associated with CES risk due to AF. Therefore, further multitrait analysis should be performed with different traits to uncover the different high-risk sources of CES. Nevertheless, our aim was to better characterize patients with CES due to AF as it is the most frequent cause of this type of stroke, for subsequently being able to find tools to detect those patients with a higher risk of developing a stroke due to an occult AF among ESUS for guiding future clinical trials with anticoagulant therapy.

In conclusion, we found and replicate 11 loci associated with CES, with eight of them having new associations. We showed that their leading SNVs are in genomic regions related to both, CES and AF, suggesting that they, together with the creation of a PRS that improves the predictive models of CES, might allow to better stratify the risk of stroke and its possible etiology to guide future clinical trials of anticoagulant therapy in AF or ESUS patients for a personalized medicine.

## Data Availability Statement

The data that supports the findings of this study are available from the corresponding author upon reasonable request.

## Ethics Statement

The studies involving human participants were reviewed and approved by Comité ético de la Fundación Docència I Recerca Mútua Terrassa. The patients/participants provided their written informed consent to participate in this study. Written informed consent was obtained from the individual(s) for the publication of any potentially identifiable images or data included in this article.

## Author Contributions

Conception and design of the work: JC-M, EM, and IF-C. Data acquisition: TS, FC, JC, JA, VO, LL-C, MF, JÁ-S, CM, MR, JJ-C, JR, LM-N, EL-C, RD-N, CV-B, GS-H, TS, LI, LH, PD, JK, RD, RD-M, LP-S, PC-R, NB, LS, RdC, JM, CC, J-ML, JM-F, and IF-C. Formal analysis and methodology: JC-M, EM, CG-F, NC, ML, and IF-C. Interpretation and supervision and writing—original draft: JC-M, EM, CG-F, NC, ML, JM, CC, J-ML, JM-F, and IF-C. All authors have been involved in drafting the article or revising it critically for intellectual content, writing—review and editing, and approved the submitted version.

## Funding

J. Cárcel-Márquez has received funding through an AGAUR Contract (Agència de Gestió d'Ajuts Universitaris i de Recerca; FI_DGR 2019, grant number 2020FI_B1 00157) co-financed with Fons Social Europeu (FSE) (https://agaur.gencat.cat). From Instituto de Salud Carlos III: E. Muiño is funded by a Río Hortega Contract (CM18/00198), M. Lledós is funded by a PFIS Contract (Contratos Predoctorales de Formación en Investigación en Salud, FI19/00309), C. Gallego-Fabrega is supported by a Sara Borrell Contract (CD20/00043) and Fondo Europeo de Desarrollo Regional (ISCIII-FEDER), T. Sobrino (CPII17/00027), and F. Campos (CPII19/00020) are recipients of research contracts from the Miguel Servet Program (https://www.isciii.es). This study has been funded by Instituto de Salud Carlos III PI15/01978, PI17/02089, PI18-01338, and RICORS-ICTUS RD21/0006/0006 (Instituto de Salud Carlos III), by Marató TV3 support of the Epigenesis study (https://www.ccma.cat/tv3/marato/), by the Fundació Docència i Recerca FMT grant for the Epigenesis project (https://www.mutuaterrassa.com), by Eranet-Neuron of the Ibiostroke project (AC19/00106) (https://www.neuron-eranet.eu), by Boehringer Ingelheim of the SEDMAN Study (https://www.boehringer-ingelheim.it), and GCAT Cession Research Project PI-2018-01 (http://www.gcatbiobank.org). GCAT was funded by Acción de Dinamización del ISCIII-MINECO and the Ministry of Health of the Generalitat of Catalunya (ADE 10/00026); and have additional suport by the Agència de Gestió d'Ajuts Universitaris i de Recerca (AGAUR) (2017-SGR 529).

## Conflict of Interest

The authors declare that the research was conducted in the absence of any commercial or financial relationships that could be construed as a potential conflict of interest.

## Publisher's Note

All claims expressed in this article are solely those of the authors and do not necessarily represent those of their affiliated organizations, or those of the publisher, the editors and the reviewers. Any product that may be evaluated in this article, or claim that may be made by its manufacturer, is not guaranteed or endorsed by the publisher.
