# Supplementary material for: A Polygenic Risk Score Based on a Cardioembolic Stroke Multitrait Analysis Improves a Clinical Prediction Model for This Stroke Subtype
Source: Front Cardiovasc Med. 2022 Jul 8;9:940696. doi: 10.3389/fcvm.2022.940696 (PMC9304625; doi:10.3389/fcvm.2022.940696)
Supplement: Supplementary file 1 [file Table_1.DOCX]

**FIGURES**

**Figure 1.** Workflow of the SNVs for the AF-2018 and MEGASTROKE-CES datasets. NaN: Not a number. SNV: Single Nucleotide Variant.

**Figure 2**. **Manhattan plot of MTAG-CES**. The X axis represents chromosome location, and the Y axis represents the minus logarithm on base 10 of p-value.

**Figure 3.** **Polygenic risk score (PRS) performance.** Panel A is a bar plot of the r^2^ for the PRS models of eight different thresholds in the training set. Panel B represents the p-value variation along the full range of thresholds evaluated in the training set. Panel C shows ROC curves and panel D Precision-Recall curves for the PRS performance in the independent test set.

**SUPPLEMENTARY FIGURES**

**Supplementary Figure 1.** GO Biological processes enriched in CES prioritized gene set.

**Supplementary Figure 2.** GO Biological processes enriched exclusively in analysis of AF associated genes independently of CES risk.

**Supplementary Figure 3.** Polygenic risk score (PRS) performance for the individual predictors. Panel A shows ROC curves and panel B Precision-Recall curves for the PRS performance in the independent test set.


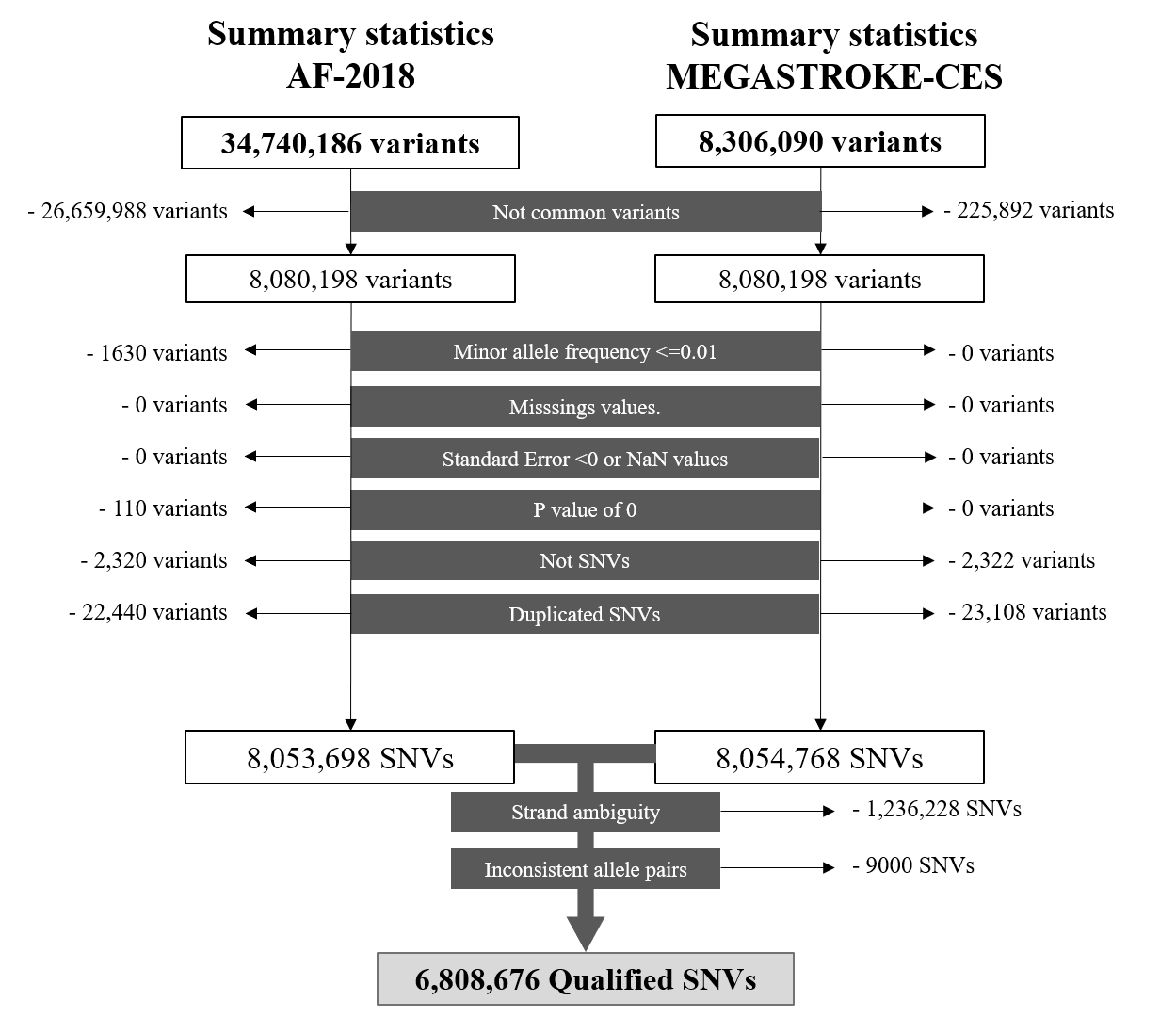


**Figure 1.** Workflow of the SNVs for the AF-2018 and MEGASTROKE-CES datasets. NaN: Not a number. SNV: Single Nucleotide Variant.

**
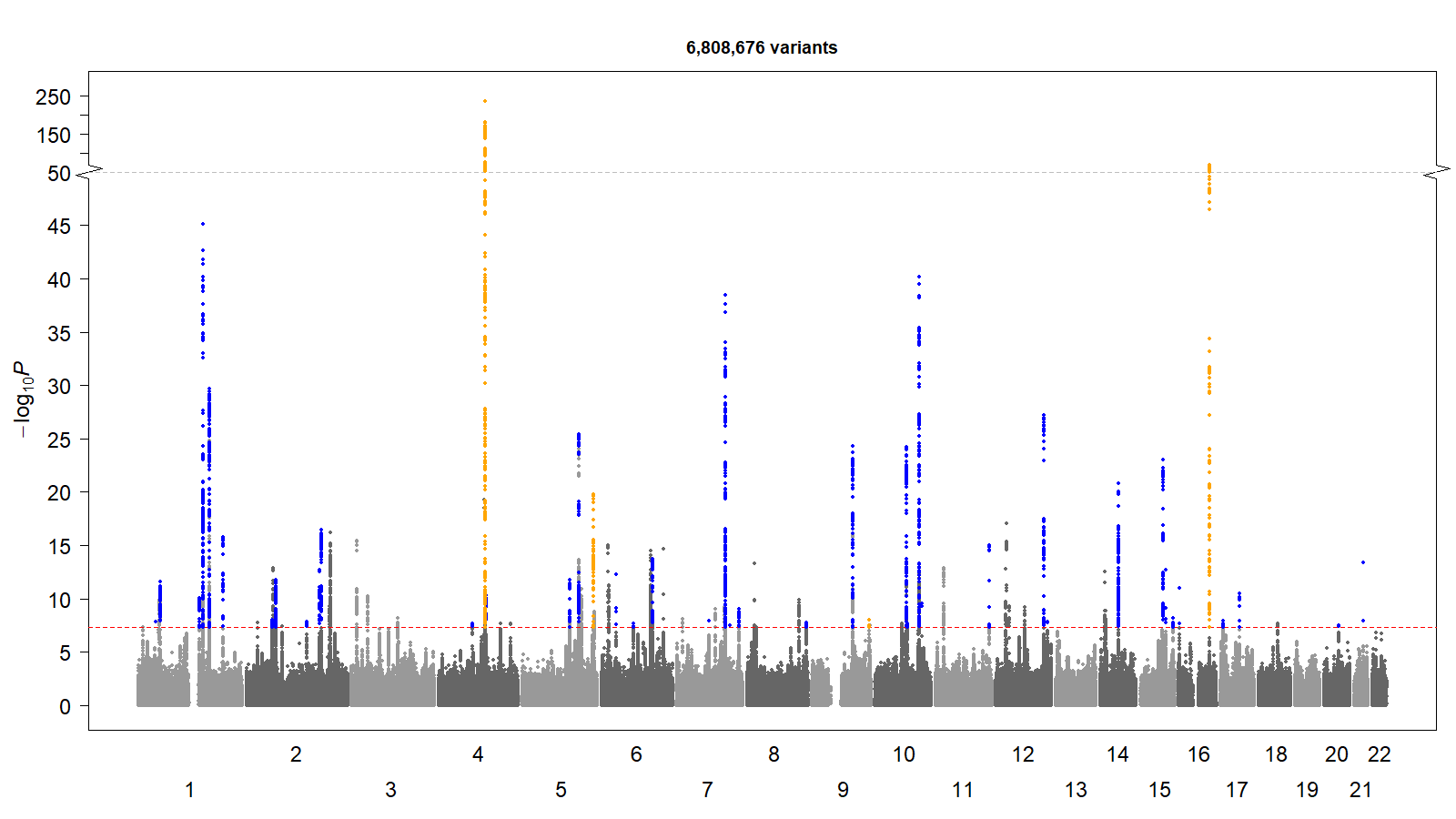
**

**Figure 2**. **Manhattan plot of MTAG-CES**. The X axis represents chromosome location, and the Y axis represents the minus logarithm on base 10 of p-value. The red line represents the GWAS-significance threshold. The novel loci are shown in blue and the established loci in yellow.

**
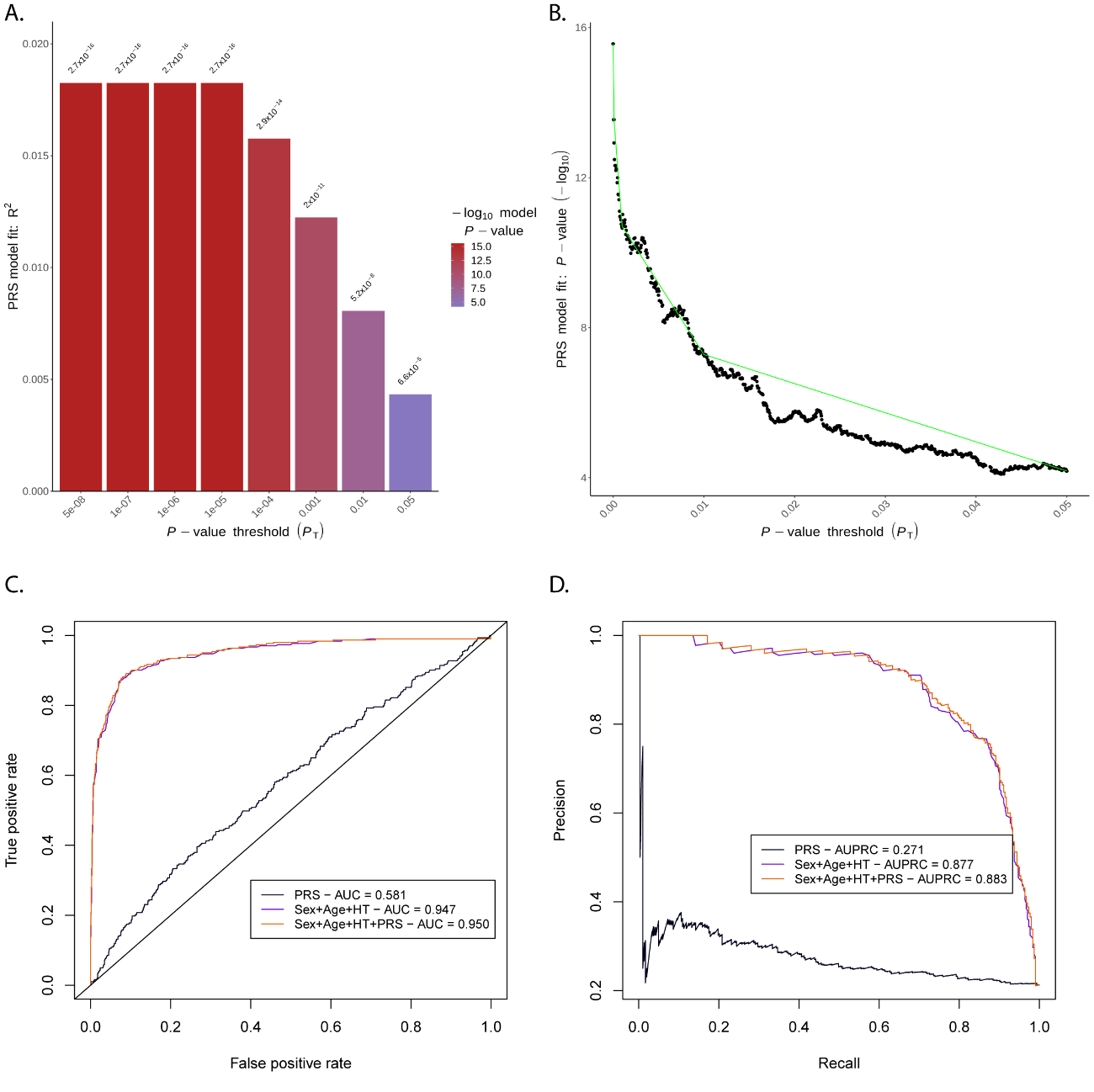
**

**Figure 3.** **Polygenic risk score (PRS) performance.** Panel A is a bar plot of the r^2^ for the PRS models of eight different thresholds in the training set. Panel B represents the p-value variation along the full range of thresholds evaluated in the training set. Panel C shows ROC curves and panel D Precision-Recall curves for the PRS performance in the independent test set. AUC: area under the ROC curve; AUPRC: area under the precision recall curve; HT: hypertension.

**
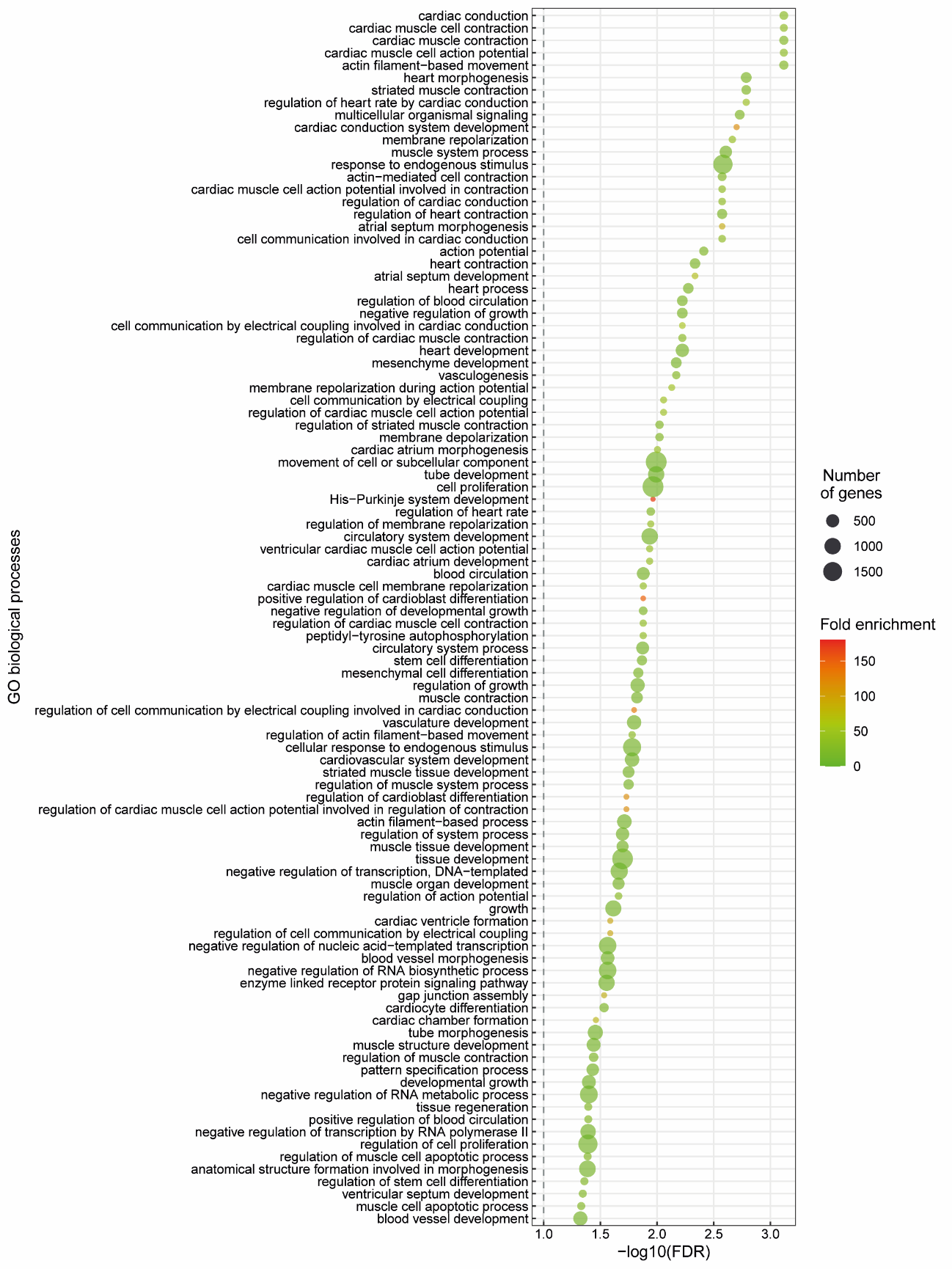
**

**Supplementary Figure 1. GO Biological processes enriched in CES prioritized gene set.**

**
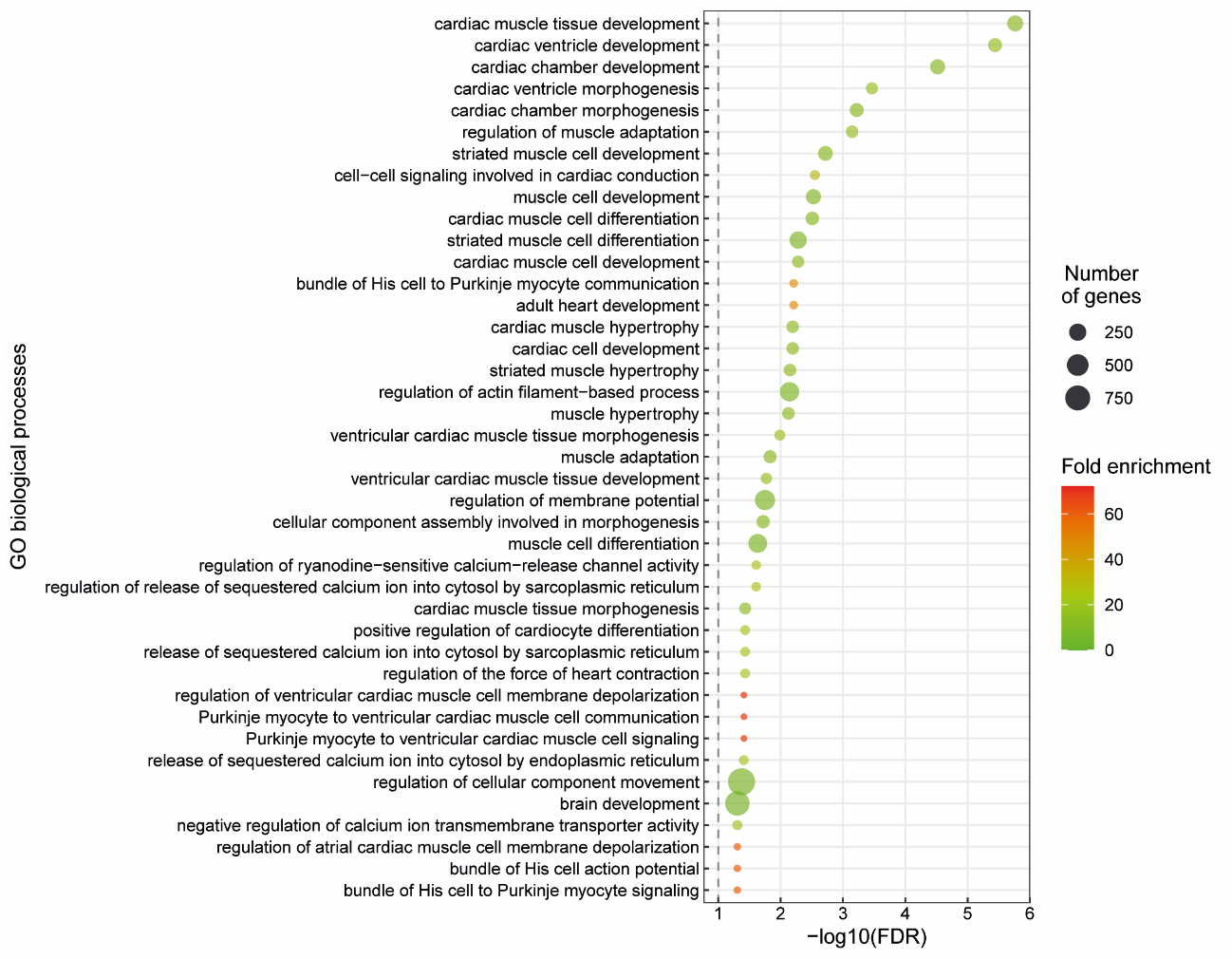
**

**Supplementary Figure 2. GO Biological processes enriched exclusively in analysis of AF associated genes independently of CES risk.**


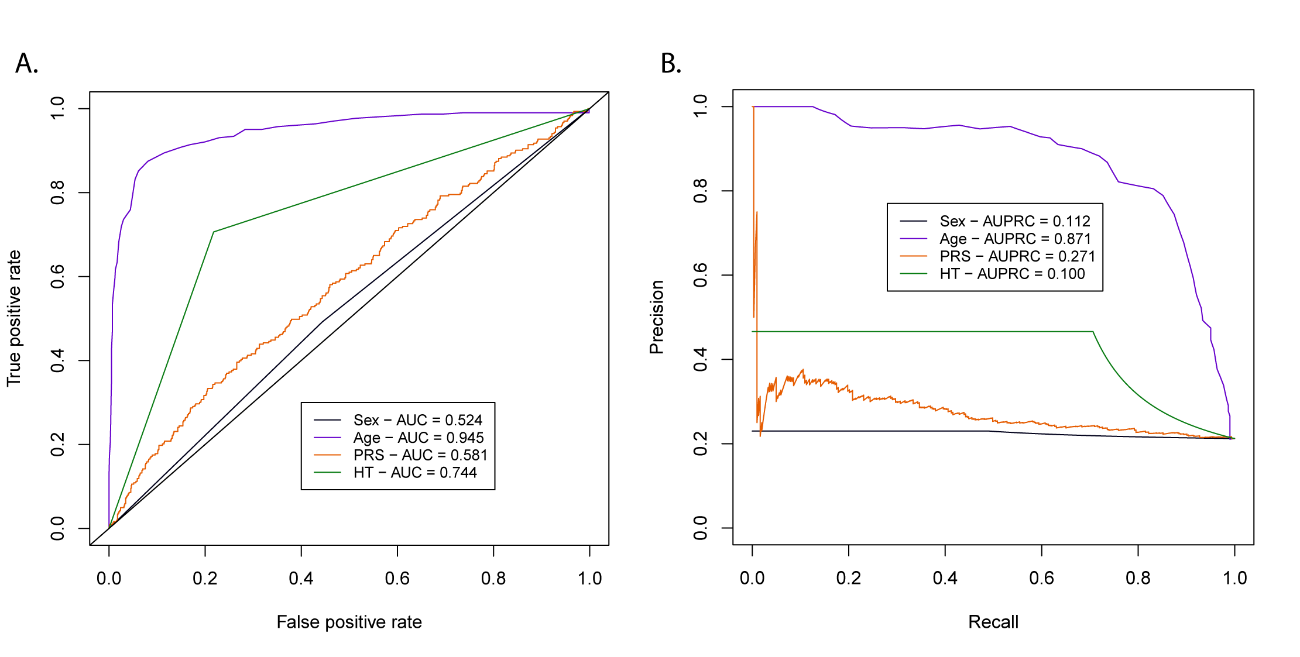


**Supplementary Figure 3.** Polygenic risk score (PRS) performance for the individual predictors. Panel A shows ROC curves and panel B Precision-Recall curves for the PRS performance in the independent test set. AUC: area under the ROC curve; AUPRC: area under the precision recall curve; HT: hypertension.
