# Supplementary material for: A Polygenic Risk Score Based on a Cardioembolic Stroke Multitrait Analysis Improves a Clinical Prediction Model for This Stroke Subtype
Source: Front Cardiovasc Med. 2022 Jul 8;9:940696. doi: 10.3389/fcvm.2022.940696 (PMC9304625; doi:10.3389/fcvm.2022.940696)
Supplement: Supplementary file 3 [file Table_3.DOCX]

Supplementary Material

# Supplementary Data

## Detailed methods

### Cohorts used in Multitrait analysis of GWAS (MTAG)

**MEGASTROKE-CES**

For the European ancestry analysis of MEGASTROKE consortium 16 different cohorts were analyzed, comprising up to 34,217 cases of ischemic stroke and 405,111 healthy controls. Stroke was defined according to the World Health Organization (WHO) as rapidly developing signs of focal (or global) disturbance of cerebral function, lasting more than 24 hours or leading to death with no apparent cause other than that of vascular origin. Strokes were defined as ischemic stroke (IS) or intracerebral hemorrhage (ICH) based on clinical and imaging criteria. IS was further subdivided into the following categories mostly using the Trial of Org 10172 in Acute Stroke Treatment (TOAST) criteria): i) large vessel ischemic stroke; ii) cardioembolic ischemic stroke; iii) small vessel ischemic stroke. Specifically, for European analysis of CES they analyzed 7,193 cases of CES and 355,468 healthy controls. Further details of the cohorts in the original publication (Malik et al., 2018).

**AF-2018 study**

A total of 60,620 cases of AF and 970,216 controls were analyzed, these patients were recruited as part of main cohorts:

HUNT.—The Nord-Trøndelag Health Study (HUNT) is a population-based health survey conducted in the county of Nord-Trøndelag, Norway from 1984 to 2009 (S Krokstad, A Langhammer, K Hveem, T L Holmen, K Midthjell, T R Stene, G Bratberg, J Heggland, 2013). They used a combination of hospital, out-patient, and emergency room discharge diagnoses (ICD-9 and ICD-10) to identify 6,493 atrial fibrillation cases and 63,142 atrial fibrillation-free controls with genotype data. Participation in the HUNT Study is based on informed consent, and the study was approved by the Data Inspectorate and the Regional Ethics Committee for Medical Research in Norway.

deCODE.—The Icelandic atrial fibrillation population consisted of all patients diagnosed with atrial fibrillation (ICD-10 code I48 and ICD-9 code 427.3) at Landspitali, The National University Hospital, in Reykjavik, and Akureyri Hospital (the two largest hospitals in Iceland) from 1987 to 2015. All atrial fibrillation cases, a total of 13,471, were included. Controls were 358,161 Icelanders recruited through different genetic research projects at deCODE genetics, excluding those in the atrial fibrillation cohort. The study was approved by the Icelandic Data Protection Authority and the National Bioethics Committee of Iceland (no. VSNb2015030021).

MGI.—MGI is a hospital-based cohort collected at Michigan Medicine, USA. Atrial fibrillation cases (n = 1,226) were defined as patients with ICD-9 billing code 427.31, and controls were individuals without atrial fibrillation, atrial flutter, or related phenotypes (ICD-9 426–427.99). MGI was reviewed and approved by the Institutional Review Board of the University of Michigan Medical School.

DiscovEHR.—The DiscovEHR collaboration cohort is a hospital-based cohort including 58,124 genotyped individuals of European ancestry from the ongoing MyCode Community Health Initiative of the Geisinger Health System, USA(Carey et al., 2016). Atrial fibrillation cases (n = 6,679) were defined as DiscovEHR participants with at least one electronic health record problem list entry or at least two diagnosis code entries for two separate clinical encounters on separate calendar days for ICD-10 I48: atrial fibrillation and flutter. Corresponding controls (n = 41,803) were defined as individuals with no electronic health record diagnosis code entries (problem list or encounter codes) for ICD-10 I48. The Study was approved by the Geisinger Institutional Review Board.

UK Biobank.—The UK Biobank is a population-based cohort collected from multiple sites across the United Kingdom(Sudlow et al., 2015). Cases of atrial fibrillation were selected using ICD-9 and ICD-10 codes for atrial fibrillation or atrial flutter (ICD-9 427.3 and ICD-10 I48). Controls were participants without any ICD-9 or ICD-10 codes specific for atrial fibrillation, atrial flutter, other cardiac arrhythmias, or conduction disorders.

AFGen Consortium.—Published atrial fibrillation association summary statistics from 31 cohorts representing 17,931 atrial fibrillation cases and 115,142 controls were obtained from the authors.

### Single Nucleotide Variants (SNVs) quality control.

A series of standard quality controls were applied to select the single nucleotide variants (SNVs) for the MTAG analysis. Variant exclusion criteria: 1) Not common to the summary statistics of the traits, 2) Minor allele frequency lower or equal to 0.01, 3) Missing values, 4) Negative standard error or not a number value. 5) P-value of 0, 6) Not SNVs, 7) Duplicated SNVs, 8) Strand ambiguity, and 8) Inconsistent allele pairs. After QCs, a total of 6,808,676 SNVs were selected (eFigure 1). Locus 15q21.3 prioritized genes *GCOM1* and *MYZAP* for AF-2018 was not evaluated due to absent of the significant SNVs of AF-2018 in the MEGASTROKE-CES analysis.

**1.1.3.** **Replication** **GWAS in GENERACION cohort**

**1.1.3.1 Study population**

We performed analysis in GENERACION study cohort (Spain). 9,105 individuals (3,479 IS and 5,626 controls). IS patients were recruited via hospital-based studies between 2003 and 2020. The participants were part of the Genetics of Early Neurological Instability After Ischemic Stroke (GENISIS), Genetic contribution to Functional Outcome and Disability after Stroke (GODS), the Genetic Study in Ischemic Stroke Patients treated with tPA (GenoTPA), the CONtrol ICtus (CONIC), and SEDMAN studies. Controls were subjects without a history of ischemic stroke, aged over 18 years, who declared they were free of neurovascular diseases before recruitment. The control cohort was collected in blood donation at primary care centers in Barcelona and in hospitals throughout Spain as a part of the GCAT, CONtrol ICtus (CONIC), Investigating Silent Stroke in hYpertensives: A magnetic resonance imaging Study (ISSYS) and the Genotyping Recurrence Risk of Stroke (GRECOS) projects. Array information, contribution of hospitals and clinical description of the cohort are present in Online Tables I-III.

The ischemic stroke patients were recruited if they had a measurable neurologic deficit on the NIHSS within 6 hours of the last known asymptomatic status, had been diagnosed with stroke by an experienced neurologist, which had been confirmed by neuroimaging and were over 18 years of age.

These patients were recruited as part of the GENISIS(Heitsch et al., 2017), GODS(Mola-Caminal et al., 2019a), and CONIC(Domingues-Montanari et al., 2010) projects.

Controls were subjects without a history of ischemic stroke, aged over 18 years, who declared they were free of neurovascular diseases before recruitment. The control cohort was collected in blood donation and primary care centers in Barcelona and in hospitals throughout Spain as a part of the GCAT(Obón-Santacana et al., 2018) (Galván-Femenia, I, 2018), CONIC(Domingues-Montanari et al., 2010), GRECOS(Fernández-Cadenas et al., 2017), and ISSYS(Riba et al., 2012) projects.

Description of the cohorts included:

GENISIS cohort.—Genetics of Early Neurological Instability after Ischemic Stroke (GENISIS)(Heitsch et al., 2017) is an international study currently recruiting patients from four different locations: United States, Finland, Poland, and Spain. The inclusion criteria for the GENISIS study are IS patients (age ≥ 18 years) Collected from 2003 to 2016 with a measurable neurologic deficit on the NIHSS within 6 hours of last known normal. Patients who received endovascular thrombectomy, or for whom consent and/or a blood sample could not be obtained were excluded. For our study we only include Spanish patients. Genotyping was performed with Human Core Exome chip (Illumina®).

GODS cohort.—Genetic contribution to functional Outcome and Disability after Stroke (GODS)(Mola-Caminal et al., 2019b) project is a study that aim find genetic factors associated to stroke outcome. All participants met the following criteria: (1) European descent, aged >18 years, diagnosis of IS in the anterior vascular territory; (2) assessed by a neurologist during the acute phase of stroke; (3) initial stroke severity >4, according to the National Institutes of Health Stroke Scale (NIHSS); (4) information on post-stroke functional status at 3 months (or alternatively between 3-6 months); (5) evidence of acute IS in a neuroimaging study; (6) lack of concomitant pathology. Individuals with stroke recurrence during the follow-up period were excluded, as well as, posterior vascular territory and lacunar strokes. Samples were genotyped at the Genetic and Molecular Epidemiology Laboratory of McMaster University (David Braley Research Institute) in Ontario, Canada, with Human Core Exome chip (Illumina®).

CONIC cohort.—CONtrol ICtus (CONIC) study(Domingues-Montanari et al., 2010) is a national study that recruited controls and IS cases participants in Vall d’Hebron Hospital between 2007 and 2008. All controls were older than 65 years of age and declared free of dementia, neurovascular and/or cardiovascular disease, as evaluated by self-description during a direct interview before recruitment. Subjects with a history of first and/or second-degree neurovascular disorder were also excluded from the study.

The IS cases were admitted to the emergency department of a university hospital who had a documented middle cerebral artery (MCA) occlusion on transcranial Doppler ultrasonography (TCD) and received tPA in a standard 0.9-mg/kg dose (10% bolus, 90% continuous infusion during 1 hour) within 3 hours of symptom onset following National Institute of Neurological Disorders and Stroke (NINDS) recommendations. Cases and controls were genotyped with Human Core Exome chip (Illumina®).

GRECOS cohort.—Genotyping RECurrence Risk Of Stroke (GRECOS)(Fernández-Cadenas et al., 2017) project is a national study that aim find genetic factors associated with the recurrence after stroke. Control participants were selected from relatives of patients (wife or husband, without any consanguinity among cases and controls) and healthy volunteers visiting the same hospital for routine testing. They were >65 years of age and classified as free of neurovascular and cardiovascular history and familial history of stroke by direct interview before recruitment. All samples were genotyped with Human Core Exome chip (Illumina®).

ISSYS cohort.—Investigating Silent Stroke in hYpertensives: A magnetic resonance imaging Study (ISSYS)(Riba et al., 2012) is an observational prospective study in hypertensive participants to determine the prevalence of silent or magnetic resonance imaging (MRI)–defined brain infarcts and cognitive impairment. This cohort comprises 1000 non-demented individuals, aged 50 to 70 years old, and diagnosed of essential hypertension at least one year before inclusion in the ISSYS study. Those individuals were genotyped with Human Core Exome chip (Illumina®).

GCAT cohort.—GCAT health databank is a collection of health data and samples from participants of the “GCAT/Genomes for Life. Cohort Study of the Genomes of Catalonia Study”(Obón-Santacana et al., 2018). The aim of the GCAT project was study the genetic and environmental factors that lead to the appearance of chronic diseases in the general population. The study is conducted in several waves of data gathering, namely GCAT1, the baseline Survey from 2014-2017 and GCAT2, the GCAT follow-up in the second year. Data collection is done with web-based self-questionnaires, direct interviews, clinical data, and analyses of DNA blood derived samples. Genome-wide genotypes have been generated using Illumina Infinium SNV-bead array technology using the Multi-Ethnic Global (MEGAEX, V.2) consortium array. We used only GCAT1 genotyped patients and we exclude individuals with heart infarct or heart diseases or with non-Caucasian ancestry.

genotPA—Consecutive Caucasian patients with acute ischemic stroke who were admitted to the emergency room and received recombinant tissue-type plasminogen activator (r-tPA) within 4.5 hours of symptom onset were recruited. Patients were enrrolled at Spanish hospitals (Vall d’Hebron University Hospital, Hospital Clinic, Hospital Universitari de Girona Doctor Josep Trueta, Hospital de la Santa Creu i Sant Pau, Hospital Universitari Germans Trias I Pujol, Hospital Universitari del Mar, Hospital de Basurto) between 2002 to 2012. The study protocol was approved by the Ethics Committee of each center, all patients or relatives signed the informed consent.

Patients were identified by medical evaluation at emergency room arrival; stroke diagnosis was performed by trained neurologists and confirmed by neuroimaging. There were no exclusion criteria regarding age, sex or ethnicity. Follow-up CT scan at 24 hours after onset of symptoms or if neurological worsening occurred were performed and was classified according to European Cooperative Acute Stroke Study (ECASS) (Larrue et al., 1997).

SEDMAN cohort. - patients ≥ 18 years old, treated with acenocoumarol or dabigatran for stroke or systemic embolism prevention following the local recommendations. All patients had a stroke or transient ischemic attack (TIA) during the previous 14 days before the initiation of anticoagulation treatment and had a diagnostic of non-valvular atrial fibrillation. Only patients with mild to moderate stroke (less than 2/3 of the vascular territory) with initial Alberta Stroke Program Early CT Score (ASPECTS) in the first CT/MRI > 6 and National Institute of Health Stroke Scale (NIHSS) < 25 were included. All patients had a general condition which allowed the 12 months’ follow-up. Only patients with stablished stroke were used in this analysis.

**Quality control and imputation**

DNA samples were genotyped on commercial arrays from Illumina (San Diego, CA) (Online Table II). Quality controls were performed using PLINK v1.9 and KING v2.1.3 software. For all datasets, samples were excluded if there was a mismatch between the genetic and reported sex, genotype call rate lower than 95%, excess or loss of heterozygosity, non-European detected as outliers of 1000 Genomes Project Phase 3 dataset (1000G), duplicate samples or relatedness at a PI-HAT>0.20. SNPs were excluded if call rate lower than 95%, located in non-autosomes, non-biallelic, strand ambiguous, monomorphic or were deviated from Hardy-Weinberg equilibrium (p-value <10^-6^ in controls, <10^-10^ in IS).

Imputation was performed in the Michigan Imputation Server Pipeline(Das et al., 2016) using Minimac4. HRC r1.1 2016 (GRCh37/hg19) was the reference panel used, with European population, and for phasing Eagle v2.4 was used. After imputation, we removed SNV with imputation score <0.6 and MAF <1%. SNVs that were not present in at least 95% of the individuals were removed.

**Samples**

DNA for all subjects were obtained from whole blood samples. A total of 10,066 samples were genotyped in the study. After QCs 9,105 samples fulfilled the QC criteria and were not missing for the phenotypes and covariates analyzed: CES, AF, sex, and age.

**Replication-stage in an independent European cohort**

*Post-analysis quality controls*

After analysis using fastGWA we removed variants with minor allele frequency < 1%, minor allele count in cases or controls < 6 and variants that deviated from Hardy Weinberg equilibrium p-value; < 1x10^-6^. Additionally, genomic inflation was estimated as lambda.

# Supplementary Figures and Tables

**
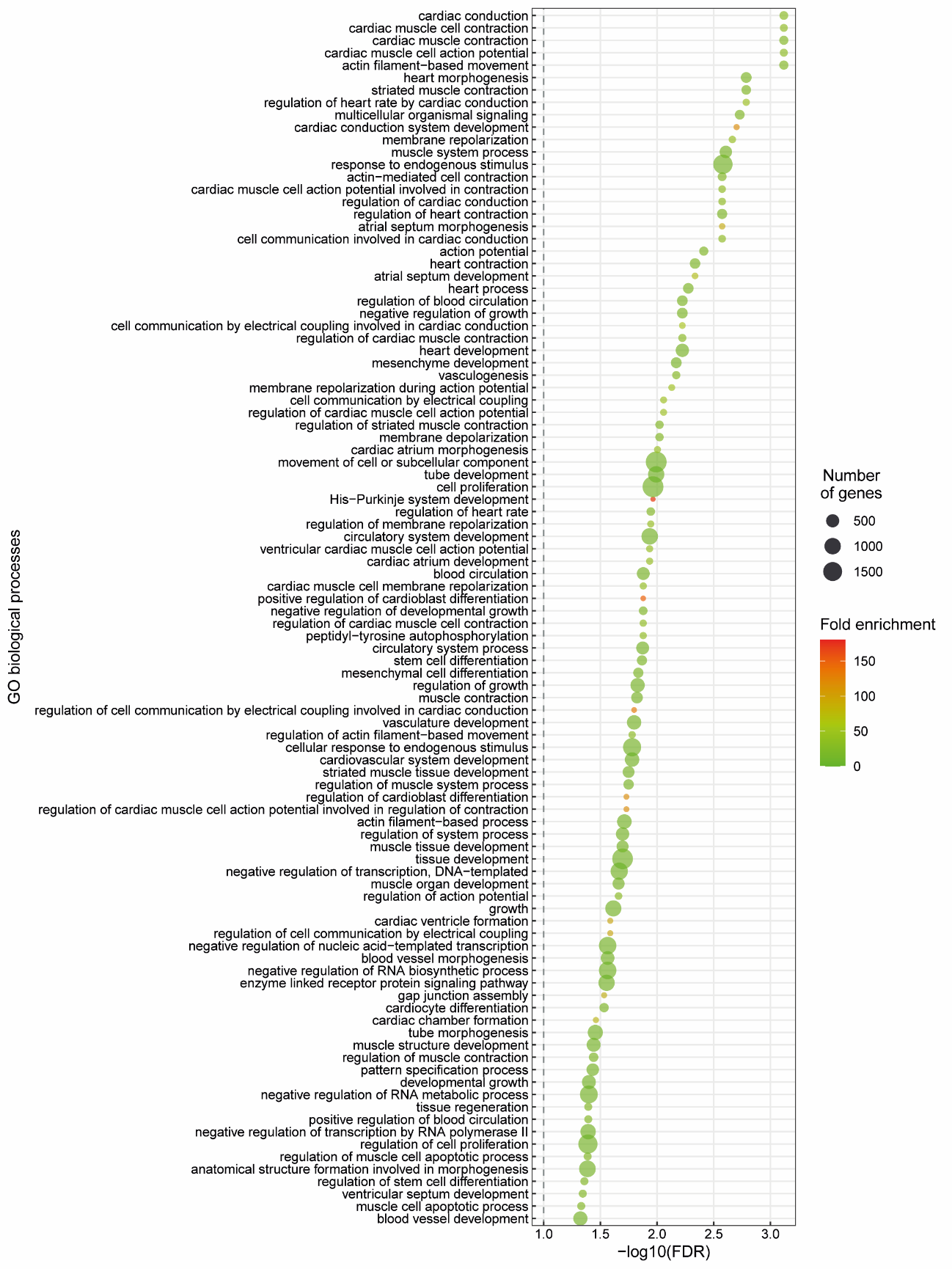
**

**Supplementary Figure 1. GO Biological processes enriched in CES prioritized gene set.**

**
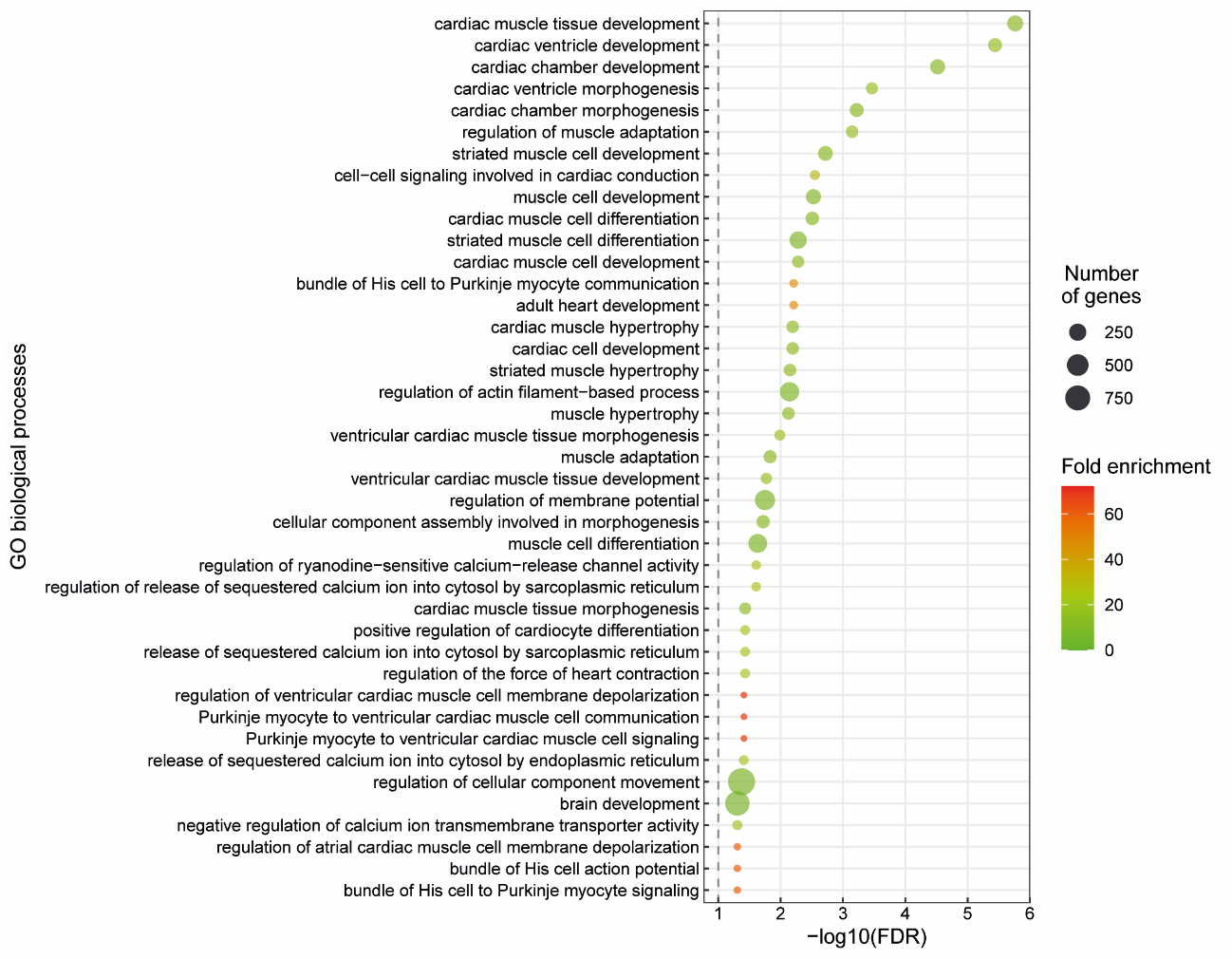
**

**Supplementary Figure 2. GO Biological processes enriched exclusively in analysis of AF associated genes independently of CES risk.**


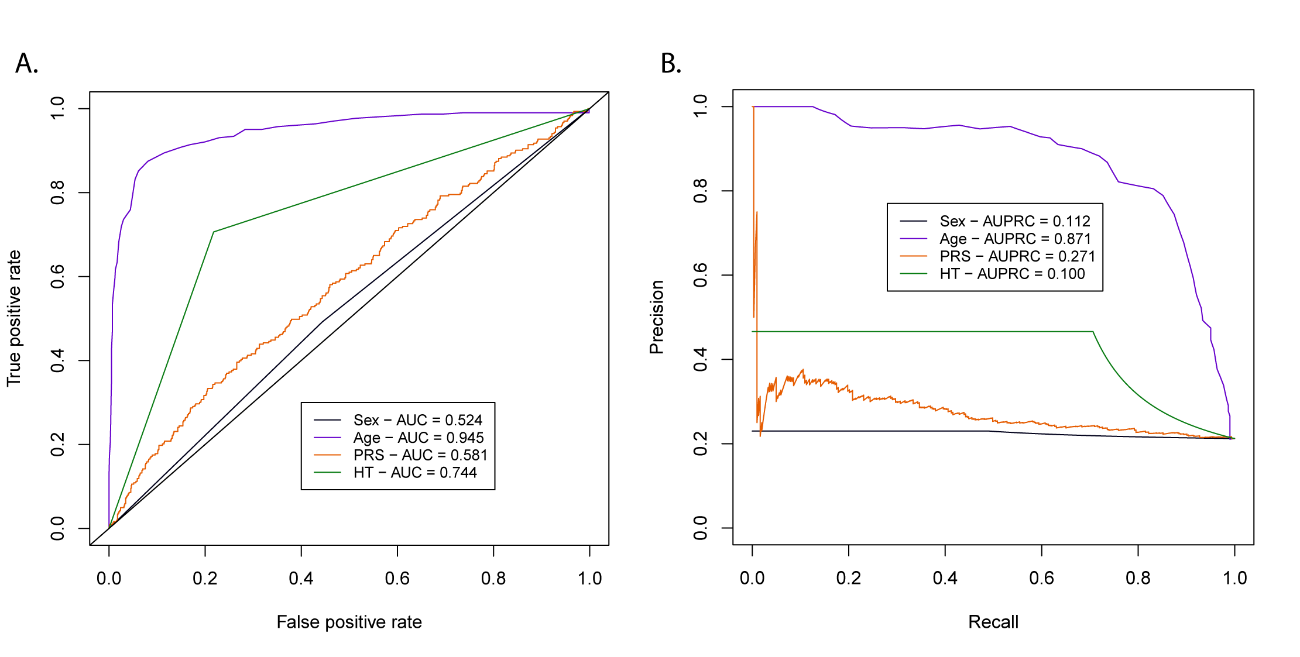


**Supplementary Figure 3. Polygenic risk score (PRS) performance for the** **individual predictors. Panel A shows ROC curves and panel B Precision-Recall curves for the PRS performance in the independent test set. AUC: area under the ROC curve; AUPRC: area under the precision recall curve; HT: hypertension.**

## Supplementary Tables

Supplementary Tables are annexed in an online data Excel file.

**Supplementary Table 1. Detailed number of individuals included form each participant Hospital on the independent cohort.**

**Supplementary Table 2. Detailed number of participants (IS and controls) included from each project**. IS: ischemic strokes patients.

**Supplementary Table 3. Clinical findings and univariate analysis of the additional cohort.**

**Supplementary Table 4. Variant-to-Gene prioritization of the 40 novel loci in the MTAG-CES analysis.**

**Supplementary Table 5. Results of CES analysis on the European independent cohort.** AA: Assessed allele, OA: Other allele, CHR: chromosome, BP: base pairs, SE: Standard Error, B: beta, Z: z-score; P: p-value. Genomic location is in Hg19

**Supplementary Table 6. Results of MTAG for previous and novel loci associated with AF**. SE: Standard Error, B: beta, Z: z-score; P: p-value.

**Supplementary Table 7. Gene Ontology of biological systems results for the gene sets of prioritized genes associated with a risk of cardioembolic stroke.**

**Supplementary Table 8. Gene Ontology of biological systems results for the gene sets of prioritized genes associated with atrial fibrillation risk exclusive.**

**Supplementary Table 9. Gene Ontology of biological systems results for the gene sets of prioritized genes associated with atrial fibrillation risk that do not overlap with those associated with CES.**

**Supplementary Table 10. Clinical characteristics of the training and test set used for the polygenic risk score analysis.**

**3. Supplementary References**

Carey, D. J., Fetterolf, S. N., Davis, F. D., Faucett, W. A., Kirchner, H. L., Mirshahi, U., et al. (2016). The Geisinger MyCode community health initiative: An electronic health record-linked biobank for precision medicine research. *Genet. Med.* 18, 906–913. doi:10.1038/gim.2015.187.

Das, S., Forer, L., Schönherr, S., Sidore, C., Locke, A. E., Kwong, A., et al. (2016). Next-generation genotype imputation service and methods. *Nat. Genet.* 48, 1284–1287. doi:10.1038/ng.3656.

Domingues-Montanari, S., Fernández-Cadenas, I., Del Río-Espinola, A., Mendioroz, M., Fernandez-Morales, J., Corbeto, N., et al. (2010). KCNK17 genetic variants in ischemic stroke. *Atherosclerosis* 208, 203–9. doi:10.1016/j.atherosclerosis.2009.07.023.

Fernández-Cadenas, I., Mendióroz, M., Giralt, D., Nafria, C., Garcia, E., Carrera, C., et al. (2017). GRECOS Project (Genotyping Recurrence Risk of Stroke). *Stroke* 48, 1147–1153. doi:10.1161/STROKEAHA.116.014322.

Heitsch, L., Ibanez, L., Carrera, C., Pera, J., Jimenez-Conde, J., Slowik, A., et al. (2017). Meta-analysis of Transethnic Association (MANTRA) Reveals Loci Associated With Neurological Instability After Acute Ischemic Stroke. in *International Stroke Conference*.

Larrue, V., Von Kummer, R., Del Zoppo, G., and Bluhmki, E. (1997). Hemorrhagic transformation in acute ischemic stroke: Potential contributing factors in the European Cooperative Acute Stroke Study. *Stroke* 28, 957–960. doi:10.1161/01.STR.28.5.957.

Malik, R., Chauhan, G., Traylor, M., Sargurupremraj, M., Okada, Y., Mishra, A., et al. (2018). Multiancestry genome-wide association study of 520,000 subjects identifies 32 loci associated with stroke and stroke subtypes. *Nat. Genet.* 50, 524–537. doi:10.1038/s41588-018-0058-3.

Mola-Caminal, M., Carrera, C., Soriano-Tárraga, C., Giralt-Steinhauer, E., Díaz-Navarro, R. M., Tur, S., et al. (2019a). PATJ Low Frequency Variants Are Associated with Worse Ischemic Stroke Functional Outcome: A Genome-Wide Meta-Analysis. *Circ. Res.* 124, 114–120. doi:10.1161/CIRCRESAHA.118.313533.

Mola-Caminal, M., Carrera, C., Soriano-Tárraga, C., Giralt-Steinhauer, E., Díaz-Navarro, R. M., Tur, S., et al. (2019b). *PATJ* Low Frequency Variants Are Associated With Worse Ischemic Stroke Functional Outcome. *Circ. Res.* 124, 114–120. doi:10.1161/CIRCRESAHA.118.313533.

Obón-Santacana, M., Vilardell, M., Carreras, A., Duran, X., Velasco, J., Galván-Femenía, I., et al. (2018). GCAT|Genomes for life: a prospective cohort study of the genomes of Catalonia. *BMJ Open* 8, e018324. doi:10.1136/bmjopen-2017-018324.

Riba, I., Jarca, C. I., Mundet, X., Tovar, J. L., Orfila, F., Nafría, C., et al. (2012). Cognitive assessment protocol design in the ISSYS (Investigating Silent Strokes in hYpertensives: A magnetic resonance imaging Study). *J. Neurol. Sci.* 322, 79–81. doi:10.1016/j.jns.2012.06.015.

S Krokstad, A Langhammer, K Hveem, T L Holmen, K Midthjell, T R Stene, G Bratberg, J Heggland, J. H. (2013). Cohort Profile: The HUNT Study. *Int. J. Epidemiol.*, 968–77. Available at: https://academic.oup.com/ije/article/42/4/968/655743 [Accessed July 10, 2020].

Sudlow, C., Gallacher, J., Allen, N., Beral, V., Burton, P., Danesh, J., et al. (2015). UK Biobank: An Open Access Resource for Identifying the Causes of a Wide Range of Complex Diseases of Middle and Old Age. *PLoS Med.* 12. doi:10.1371/journal.pmed.1001779.
